# “*Sleepless nights are a daily reality for us*” how women experiencing homelessness in Addis Ababa, Ethiopia describe street life: a photovoice study

**DOI:** 10.3389/fpubh.2025.1488770

**Published:** 2025-02-05

**Authors:** Kalkidan Yohannes, Mats Målqvist, Hannah Bradby, Yemane Berhane, Dagmawit Tewahido, Sibylle Herzig van Wees

**Affiliations:** ^1^Department of Women’s and Children’s Health, Centre for Health and Sustainability, Uppsala University, Uppsala, Sweden; ^2^WOMHER: Women’s Mental Health During the Reproductive Lifespan, Interdisciplinary Research Centre, Uppsala University, Uppsala, Sweden; ^3^Department of Psychiatry, Dilla University, Dilla, Ethiopia; ^4^Department of Sociology, Uppsala University, Uppsala, Sweden; ^5^Addis Continental Institute of Public Health, Addis Ababa, Ethiopia; ^6^Department of Global Health and Population, Harvard T.H. Chan School of Public Health, Boston, MA, United States; ^7^Department of Global Public Health, Karolinska Institute, Stockholm, Sweden

**Keywords:** photovoice, participatory research, women’s homelessness, rooflessness, qualitative research, Ethiopia, East Africa

## Abstract

**Introduction:**

Homelessness among women of reproductive age is a global problem. Several unique gender-based issues affect homeless women’s well-being, including reproductive health issues, their homelessness experiences, and a high rate of sexual violence. In this study, we aimed to describe women’s experiences of street homelessness in their own terms and their suggestions to address their unmet needs.

**Methods:**

This photovoice study draws on photos, focus group discussions, and semi-structured interviews. We conducted the study in collaboration with women in their reproductive years experiencing homelessness (*n* = 9). A total of 80 photos were taken, and 40 were chosen to be discussed in interviews and further focus group discussions. The participating women selected photographs, explained their significance, and codified them based on how they related to their lives. Data from these discussions were then analysed using a reflexive thematic approach.

**Results:**

Four themes were developed from the data: (a) deprivation of basic needs; (b) experiencing dependency, shame, and seclusion while dealing with the burden of street life; (c) the vulnerability and neglect of children; and (d) being resilient to harsh conditions. In this study, women’s street life was characterised by numerous unfavourable aspects, including unmet needs, human rights violations, social exclusions, substance use, and child protection issues. Participants provided suggestions for change and confirmed their belief that adequate housing represents one of the most urgent unmet basic needs of people experiencing homelessness. They also emphasised the critical need for employment opportunities, non-discriminatory provision of social support, treatment programs for substance misuse, and legal and social protection.

**Conclusion:**

This study contributes to understanding how women experiencing homelessness describe and articulate their living circumstances and what they perceive needs to be addressed. Based on participants’ proposal for change, comprehensive services are needed to address women’s multifaceted issues. However, the mitigation strategies and long-term effects of women’s homelessness require further research.

## 1 Introduction

Homelessness is a multifarious global phenomenon that affects physical, social, spiritual, legal, and territorial (privacy) aspects of an individual’s life (1, 2). Although defining and quantifying homelessness remains a challenging task, estimates suggest that approximately 2 percent of the global population is homeless, with street homelessness among women on the rise, mirroring the trend among men (3, 4). The inability to provide essential services, safety, and security for these people is a violation of human rights (1) and of the International Covenant on Economic, Social, and Cultural Rights (ICESR) (5), which 172 countries, including Ethiopia, have signed.

All forms of homelessness affect all population groups, regardless of gender, in every country (1). However, women experiencing homelessness face multifaceted and unique issues on top of the problems that both men and women face (6). These include challenges related to raising children (7), reproductive health issues (8), and intimate partner violence (9). This study focuses on women experiencing street homelessness in Addis Ababa, Ethiopia, who sleep in public spaces such as parks, by railway embankments, under bridges, beside roadways, and on riverbanks.

Studies have investigated the influence of street life on women’s lives, their wellbeing, and their “invisibility” in high-, middle-, and low-income countries (6, 10, 11). Mayok et al. (12) reported the challenges of women’s homelessness in Europe. Some studies have also revealed situations of women’s homelessness in different parts of Africa, specifically in Ghana (13), Nigeria (14), and Ethiopia (15, 16). Critical concerns for women include rape, violent victimisation, unplanned pregnancies, and involvement in crimes, prostitution, and a severe lack of maternity services. Even though women are exposed to numerous specific problems because of their gender (6, 12), studies have shown that women experiencing homelessness use a variety of coping strategies that help them leave the streets (16, 17). For example, they show optimism (17), confront problems (18), rely on social networks (19), and use faith as both a coping mechanism and an influencing factor to avoid difficulties (20). In contrast to this, women experiencing homelessness and those who were using substances employed emotion-focused defence mechanisms such as preoccupations with various addictions (21, 22). Those previously at risk would be exposed to an unhealthy avoidant coping style in which a lower use of problem-focused coping was associated with poorer mental health (23). Another study showed that women with dependent children cope with stress by internalising it, which could result in more mental health issues than childless women have (24).

In Ethiopia, a qualitative study was conducted among 31 women experiencing homelessness (16). The authors reported that begging and child prostitution were used as coping mechanisms.

While existing studies highlight the significant issue of homelessness in Addis Ababa, Ethiopia, it primarily relies on researchers’ interpretations. No attention has been given to how women experiencing homelessness see, perceive, represent, and personalise their experiences of street homelessness. There is a lack of research on understanding these women’s suggestions and proposals for change. In other words, their perspectives, the often-invisible aspects of homelessness, and the specific issues that need to be addressed to improve their lives in Addis Ababa from their points of view remain poorly understood. Therefore, we aimed to conduct a photovoice study. Community-based participatory research methods such as photovoice can help us understand community needs and make positive individual and community changes (25, 26). Photovoice is a participatory health promotion strategy that allows individuals to identify, represent, and enhance their community through photography (25). This method is appropriate for understanding the living conditions of individuals experiencing homelessness and the meaning they attribute to their lives (27, 28). The photovoice method has been used, in addition to other methods, to understand women’s experiences of homelessness in the United States (29), Canada (30), and New Zealand (31). Using the Photovoice method, this study aimed to understand how women experiencing homelessness in Addis Ababa, Ethiopia, describe their experiences of street homelessness in their own terms and their suggestions to address their unmet needs.

## 2 Methodology

### 2.1 Study setting and period

We conducted the fieldwork of this study in Addis Ababa, the capital of Ethiopia, in December 2023. The population of Ethiopia was over 126 million in 2023 (32), making it the second most populous nation in Africa and the twelfth most populated country in the world (32). Ethiopia has experienced rapid urbanisation, which has multiple implications for sustainable development (33). There is no precise data regarding the magnitude of women experiencing street homelessness in Ethiopia; however, the city administration estimated that around 50,000 people experience homelessness in Addis Ababa (34). Although the Ministry of Women and Social Affairs (MoWSA) is responsible for individuals experiencing homelessness, several governmental and non-governmental organisations provide different support and services for them (35). One of these organisations is the Birhan Integrated Community Development Organisation (BICDO), where participant recruitment occurred. This organisation was established through government collaboration in Addis Ababa, Ethiopia, to support mothers and their children experiencing street homelessness. Assistance includes transitional housing, training, and a grant to start a business.

### 2.2 Study design

We conducted a photovoice study, a community-based participatory action research approach using photography (25). In the early 1990s, Wang and Burris (25, 36) developed this action research method to help people identify, represent, and enhance their communities. This method encourages individuals to document and reflect on their experiences and ideas through photographs to create conversations about communities, reach policymakers, and promote critical dialogue (25). This process allows people to become involved in the research process and develop a sense of responsibility for the community (25, 26). Photovoice reduces language and traditional communication barriers that prevent group members from expressing their concerns (25). This study was guided by the COREQ (Consolidated Criteria for Reporting Qualitative Research) (37) and the Braun and Clarke (38) 15-point Thematic Analysis Checklist.

### 2.3 Study participants

We collaborated with women aged 18–49 who had been experiencing street homelessness in Addis Ababa for at least 6 months before the study and were willing to participate. Participants included women from Addis Ababa and parts of Ethiopia, including the Amhara, Oromia, and central Ethiopia regions. We excluded street-living mothers who were mentally and physically unwell, under the influence of alcohol or other substances, or unable to speak Amharic during data collection.

### 2.4 Sampling and recruitment

This study was conducted in collaboration with women experiencing homelessness who received various training and financial support from the BICDO. We selected participants based on convenience sampling technique. Together with two social workers at BICDO, we recruited 10 participants.

### 2.5 Training on the photovoice method

**Figure 1:**
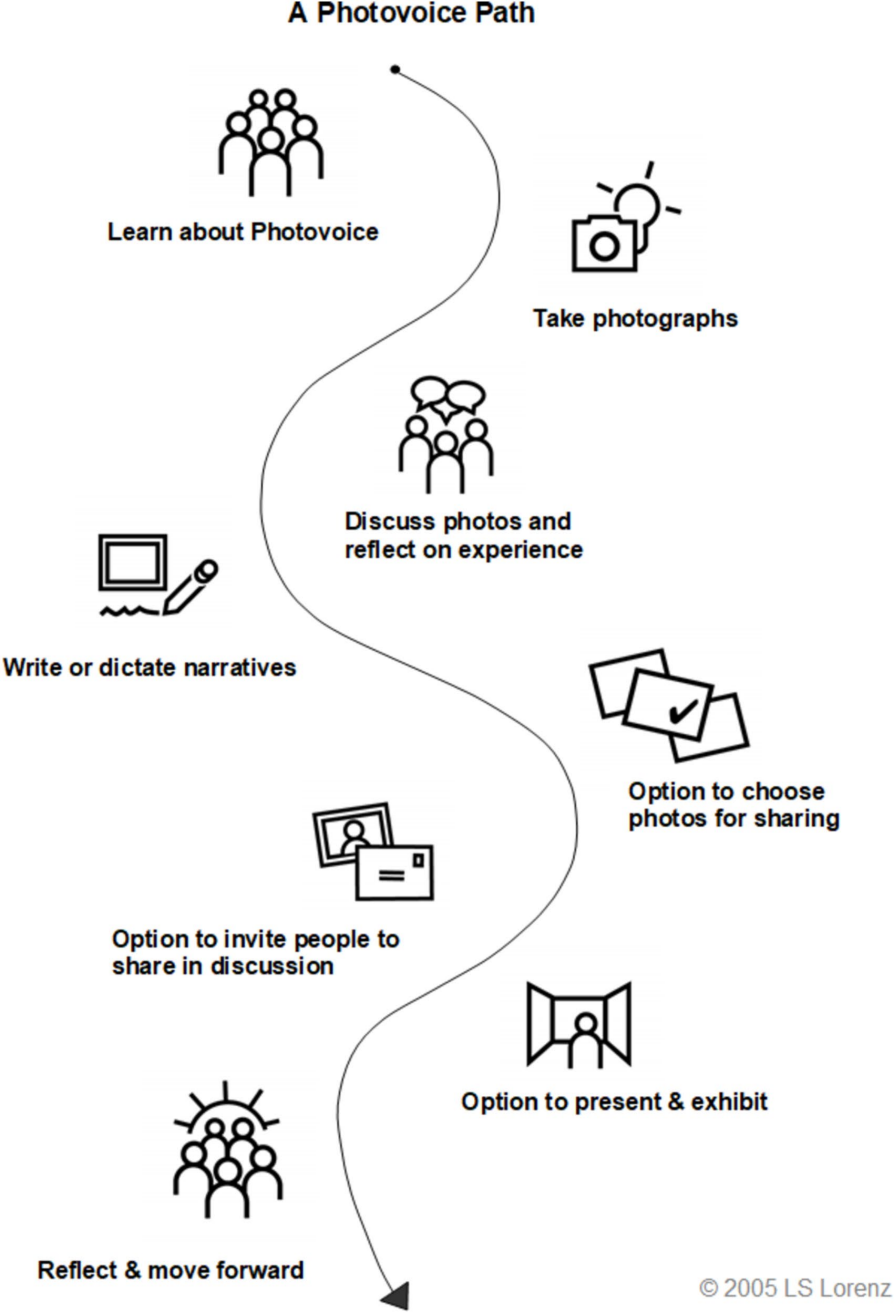
A photovoice path. Source: Lorenz (39).

The first author (KY) delivered the training on photovoice. The first part of the workshop covered the study’s objective, what a photovoice study is, the role the women would play in the research, the ethical issues associated with photographs and their involvement in the project, and the procedures to follow using a photovoice path, which was developed by Lorenz (39) (Figure 1).

Then, KY gave the women three days to think over the pros and cons of participating in the study and to communicate with others about whether they wanted to enrol in the project. KY and the women met for the second workshop on Monday, December 11, 2023. Seven women agreed to participate in the project, and three women declined. The reasons not to participate were either related to health and administrative issues or were not disclosed. Consequently, we recruited two additional women. In the second workshop, KY provided basic technical information about disposable cameras and explained how to feel comfortable using the camera, achieve the desired results, and enjoy the photography exercise. During this phase, KY informed the participants about the process during and after the fieldwork, including what would happen to all the photos and the practical and ethical issues surrounding the fieldwork.

### 2.6 Data collection

**Figure 2:**
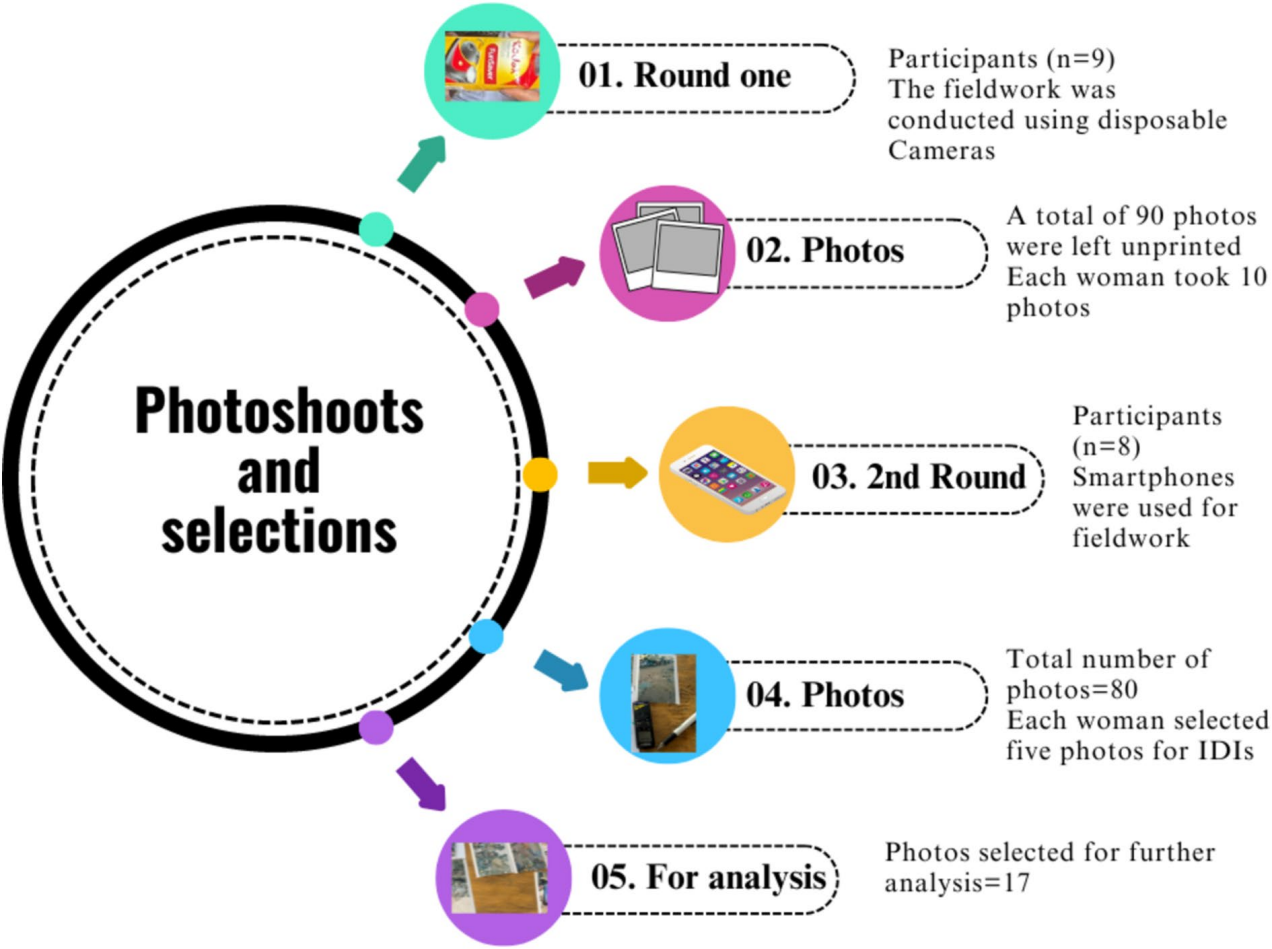
Fieldwork and photo selection process. Graphics: adapted from https://www.canva.com/.

We used five Kodak Fun Saver single-use disposable cameras and two audio recorders for the study. Nevertheless, printing options were limited due to a lack of chemicals used to print photographs taken with disposable cameras. Consequently, KY gave a smartphone to the participants for the second round of photoshoots, and each woman took turns taking photos. Although one participant could not join the second round of smartphone photography, she could engage in a discussion group with eight other participants. Participants submitted photos two days before their scheduled individual and group sessions. The figure below illustrates the selection of photos and the fieldwork process (Figure 2).

### 2.7 In-depth interviews

**Figure 3:**
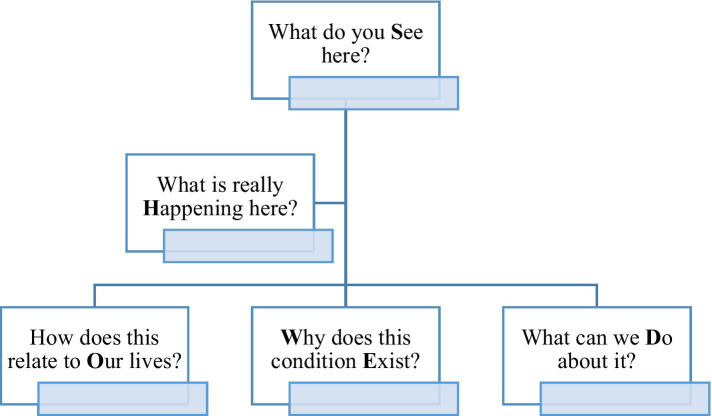
The SHOWeD technique.

KY conducted eight semi-structured interviews. All interviewees were mothers, so their children occasionally interrupted the interviews. Interviews were audio-recorded. Participants were asked to choose five photos that best represented street life according to their perceptions before the interview. KY conducted semi-structured interviews using questions that articulated the mnemonic SHOWeD (Figure 3), initially introduced by Wallerstein and Bernstein (40). These questions helped us understand each image’s relation to the study’s objective.

### 2.8 Focus group discussions

**Figure 4:**
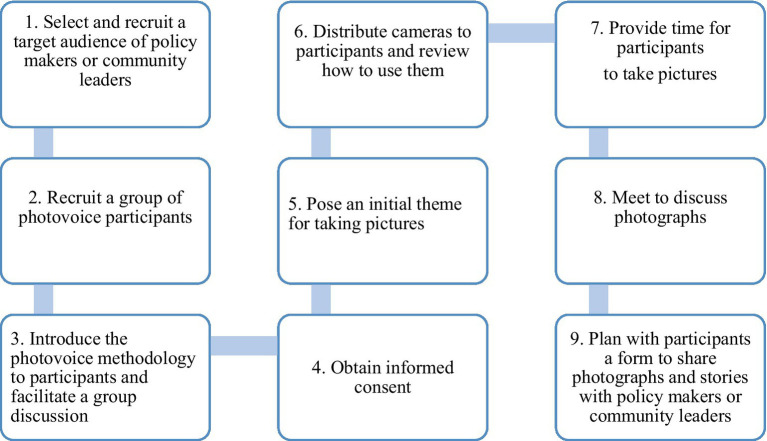
Steps for conducting a photovoice study [adapted from Wang and Mary Burris (76)].

We conducted two focus group discussions (FGDs) to review specific images, discuss photos, create categories, collaborate, and develop collective categories in a group setting. The first FGD lasted 46 min, and the second lasted 2 hrs. The overall photovoice process followed in this study is outlined in Figure 4.

### 2.9 Data quality management

During the training, KY introduced ethical considerations for photography and fieldwork and the personal and interpersonal skills necessary for gathering photovoice information. KY translated the SHOWeD interview guide into Amharic and then returned it to English as part of the data. She informed the participants of their responsibilities before they entered the fieldwork stage. As part of the overall supervision of the study, a senior researcher, SHvW, regularly communicated and audited the participatory research format and application, and KY operated as the photovoice facilitator and oversaw the photovoice progress daily to ensure accuracy, completeness, and appropriate data management and storage. Following the European General Data Protection Regulation (GDPR), we handled all data. We limited data access to the research team and ensured that sensitive information and documents would not be disclosed to others. The research database was kept under strict security conditions, and only research team members had access to photos and paper-based documentation, such as consent forms, which were collected from participants. Our study included certain data types (photographs) that are challenging to anonymise effectively. As a result, audio data and sensitive images were kept in a secure location (password locked), with access limited to involved researchers who should be contacted with questions.

### 2.10 Data analysis

Photovoice participants took part in a three-stage process to establish the foundation for analysis, as described by Wand and Burris: selecting photos, contextualising, and categorising (25). First, the participants selected the most critical photos from all the ones they had taken. Then, a second stage of storytelling was conducted. KY documented participants’ stories and took notes in individual and group discussions. Through KY’s facilitation, the women moved on to the third phase, codification. The participants interpreted the images based on their perspectives and experiences with the facilitator’s assistance. After completing these stages with the participants, KY and SHvW conducted analysis of the audio files, transcriptions, and memos/field notes following the Braun and Clarke’s approach to reflexive thematic analysis (41).

### 2.11 Reflexivity

As a PhD student and the primary investigator, KY has taken a reflexive approach to the research process and her role: she has questioned and highlighted her central assumptions. KY’s Ethiopian heritage, her previous experience of living in Addis Ababa, and the fact that she is in her reproductive years all have influenced her perspective on the living conditions of the women on the city streets. Although these factors may have influenced her facilitation of this study, which was carried out in collaboration with women experiencing rooflessness, she positioned herself as an outsider due to her lack of experience working with organisations providing services in this area. The fact that KY is a psychiatric professional, similar to the participants in terms of culture, gender, language, customs, and values, has helped her understand and manage their behaviour, enabling her to facilitate group interaction. Besides, it allowed her to provide reassurance and create a non-judgmental environment for those with communication problems. However, having a psychiatric background may have also led her to interpret their emotions, speeches, and behaviour in an overly empathetic manner. Aside from the principal investigator, three women and two men with extensive qualitative research expertise were overseeing the study, including assistant professors in development studies (SHvW) and public health (DT), as well as professors in epidemiology and public health (YB), global health (MM), and sociology (HB). Ethiopians DT and YB have conducted extensive research in the country. In this setting, SHvW has also conducted research.

The research team did not have any prior association with participants or the organisation involved, thereby reducing bias in the selection of participants, the implementation/facilitation of fieldwork, and the organisation of the study. While analysing the data, KY had to revise her previous understanding of legal protection. Having assumed that the police would protect the public, when the participants reported their conditions, she was left wondering: Who is responsible for protecting the street dwellers from the police and controllers? Do homeless individuals have the right to build shelters in public areas? KY encountered these seemingly contradictory ideas while analysing the data. Apart from its role as a community-based participatory method, the photovoice approach helped us prevent power imbalances between researchers and participants, allowing the latter to speak for themselves.

In summary, the data was analysed thematically under SHvW’s supervision. In addition to SHvW’s extensive experience in qualitative analysis, the frequent meetings throughout the study have substantially reduced the chance of bias that KY might introduce’ preconceived notions.

### 2.12 Trustworthiness

Interdisciplinary co-authors with experience in social science and health cross-reviewed the data and interpreted it. We gathered data from multiple sources to gain a more comprehensive understanding of the street life of women experiencing homelessness. This included photographs taken along the street that reflect the women’s living circumstances and interviews/focus group discussions with the women.

## 3 Findings

Nine women between the ages of 18 and 49 participated in the study. Each woman had at least one child, and the maximum number of children per woman was three. Seven participants were single or separated, and two were widows. Five participants had primary education or higher, whereas four had no formal education. There was a difference between the length and the level of chronicity of street rooflessness among the women; however, all of them had lived on the streets at some point in their lives. The most common form of street homelessness was chronic homelessness (those who have been homeless for more than a year or have experienced rooflessness regularly), which seven women had experienced. Some had been homeless for more than 5 years, while others had been on the streets for less than a year. However, there were no translationally homeless women in the study (short-term homeless, usually less than a month). During data collection, all participants received assistance from the BICDO (Table 1).

**Table 1:** Sociodemographic characteristics of study participants in Addis Ababa, Ethiopia, 2023 (*n* = 9).

Variables	Categories	Frequency
Age in years	18–25	1
	26–29	6
	30 and above	2
Marital status	Single, divorced, separated	7
	Widowed	2
Number of children	Having only one child	1
	Having two or more children	8
Level of education	No formal education	4
	Primary education	4
	Secondary education and above	1
Homelessness duration	Chronically homeless (> a year and above)	7
	Short-term homeless (6 months to 1 year)	

### 3.1 Themes

**Figure 5:**
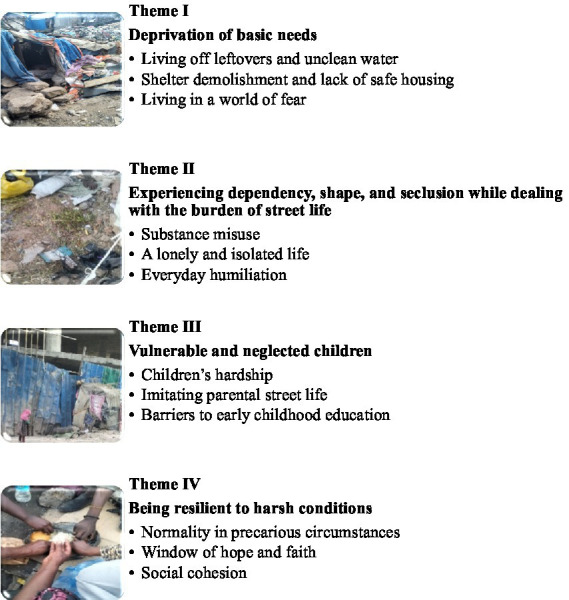
Four themes and 12 sub-themes emerged from the analysis.

The final analysis consisted of 17 photos and narratives selected from an initial pool of 80 photos. The following four themes emerged from twelve sub-themes (Figure 5).

#### 3.1.1 Theme I: deprivation of basic needs

This theme focuses on the unmet needs of people experiencing homelessness, living circumstances, and abandoned areas. Using photos, participants illustrated homeless individuals’ unmet needs, such as physiological and emotional, as well as safety and security concerns. The categories below illustrate how unmet basic needs and emotional threats exist in women’s street life experiences and living circumstances.

##### 3.1.1.1 Living off leftovers and unclean water

**Figure 6:**
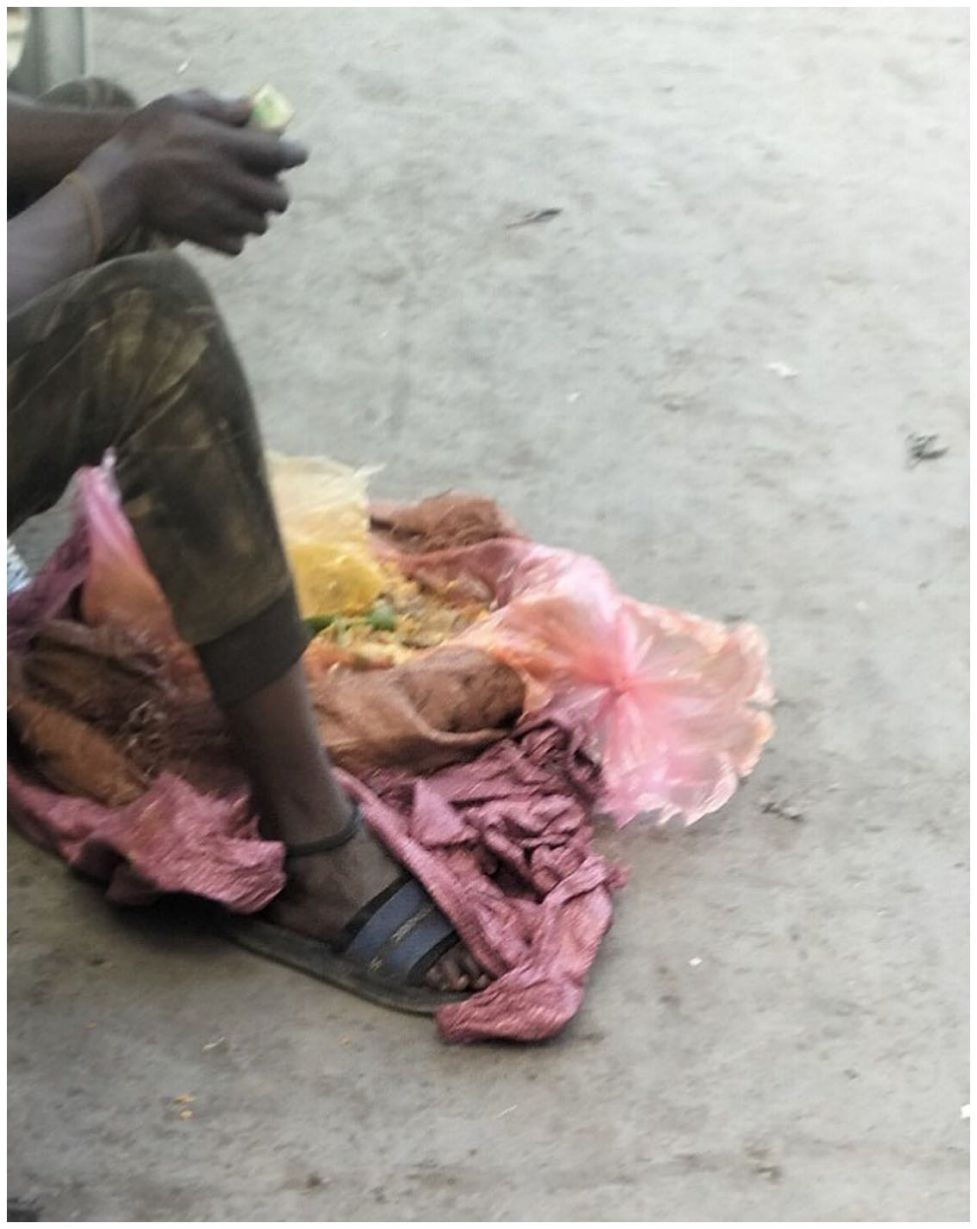
Primary food source - Bulle (Amharic: ቡሌ). Addis Ababa, Ethiopia, December 15, 2023. A man sells bulle, or leftovers, to homeless individuals around Merkato. My children and I depend on this type of food for survival. I neither cook nor purchase injera (እንጀራ) (Ethiopian food made of teff). Instead, I always sit next to them (street vendors) and buy leftovers. We do not have our mothers’ house where we can go and knock on the door and eat (Image and quote credited to a 28-year-old mother experiencing street homelessness).

Data analysis highlights that the primary food sources of participants are leftovers from hotels, restaurants, cafés, and juice and vegetable shops. Street vendors purchase and sell “bulle,” leftover food from restaurants, to people experiencing street homelessness. Getting food for free from restaurants was arduous. A participant photographed the leftover food - a picture of a street vendor selling leftover food in plastic bags on the street (Figure 6):

Participants expressed concerns about the potential health risks associated with leftover food consumption. They often use leftover food disposed of in trash bins or insanitary areas, leading to common stomach problems.

*When we eat it [leftovers], we typically experience stomach cramps and diarrhoea. However, we always use leftovers because they are inexpensive compared to other food sources. We prioritise the affordability of leftovers over any potential health risks (Interview #2, a 26-year-old mother experiencing street homelessness).*

In addition to bulle, several participants mentioned foraging fruit peels and expired supermarket foods from waste bins as potential food sources. Although these food sources are unsafe, the women stress that they must survive regardless of any harm they may suffer.

A participant described this as follows:

*My kids and I ate the leftover food that we picked up from the trash. I also picked up the discarded chicken meat from the waste disposal area a few days before it expired and cooked it for them. If the public throws around rat poison, we could pick it up and consume it. This is a risky situation. (Interview # 1, a 30-year-old mother experiencing street homelessness).*

**Figure 7:**
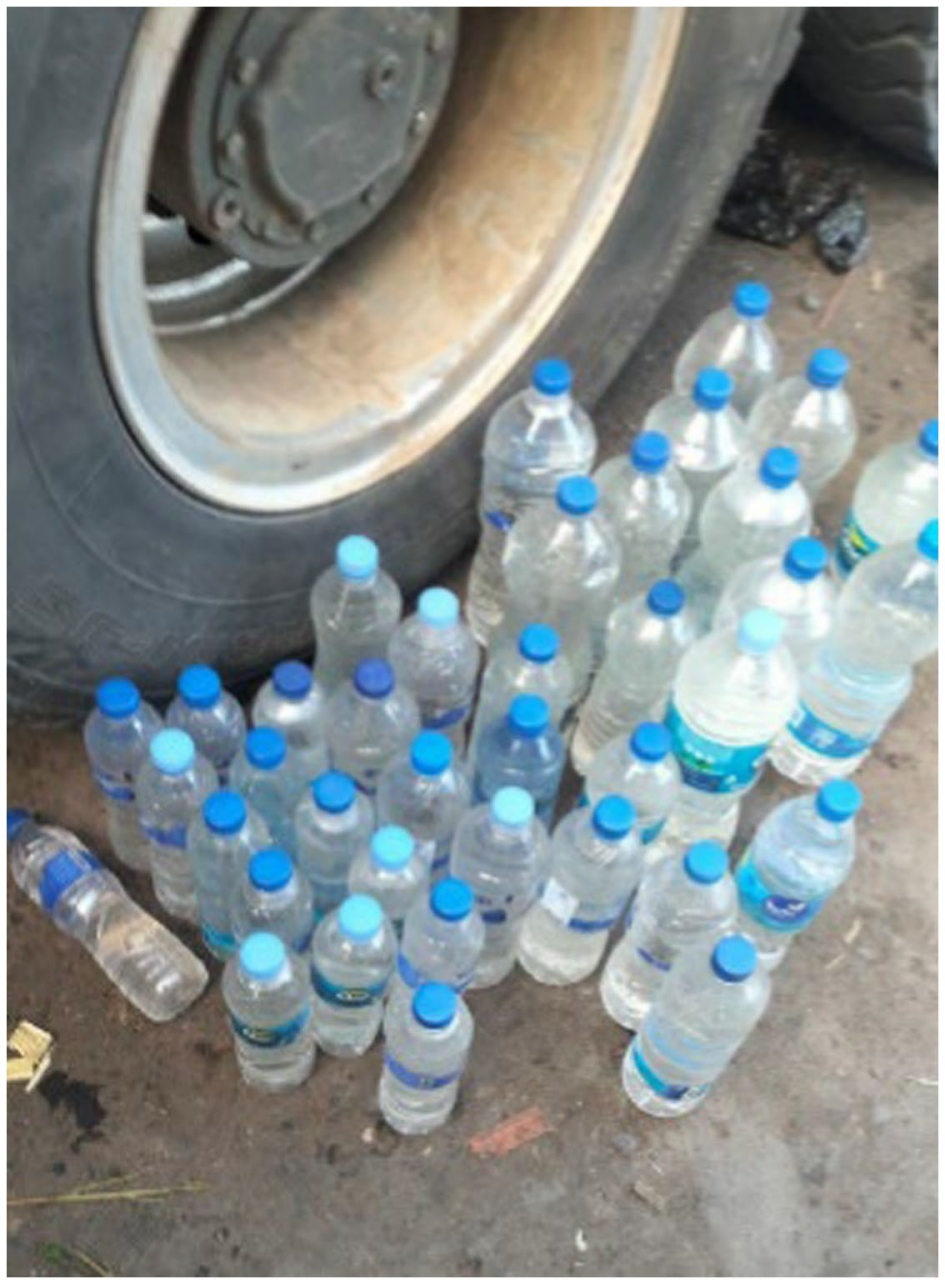
A drinking water source for individuals experiencing street homelessness. Addis Ababa, Ethiopia, December 15, 2023 (Image credited to a 27-year-old mother experiencing street homelessness).

In addition to food scarcity, participants had difficulty accessing drinking water, washing their clothes, preparing food, and maintaining hygiene (Figure 7).

Street vendors in the city sell water from various sources, including unclean sources, for 2–10 Birr per litre (0.012–0.12 US dollars).

##### 3.1.1.2 Shelter demolishment and lack of safe housing

Participants’ shelters are made of cardboard, tarps, plastic, stones, and wood in waste-filled and underserved areas. They emphasised that uncertainty regarding how and when controllers will demolish these shelters results in a constant fearful living situation for everyone on the streets.

**Figure 8:**
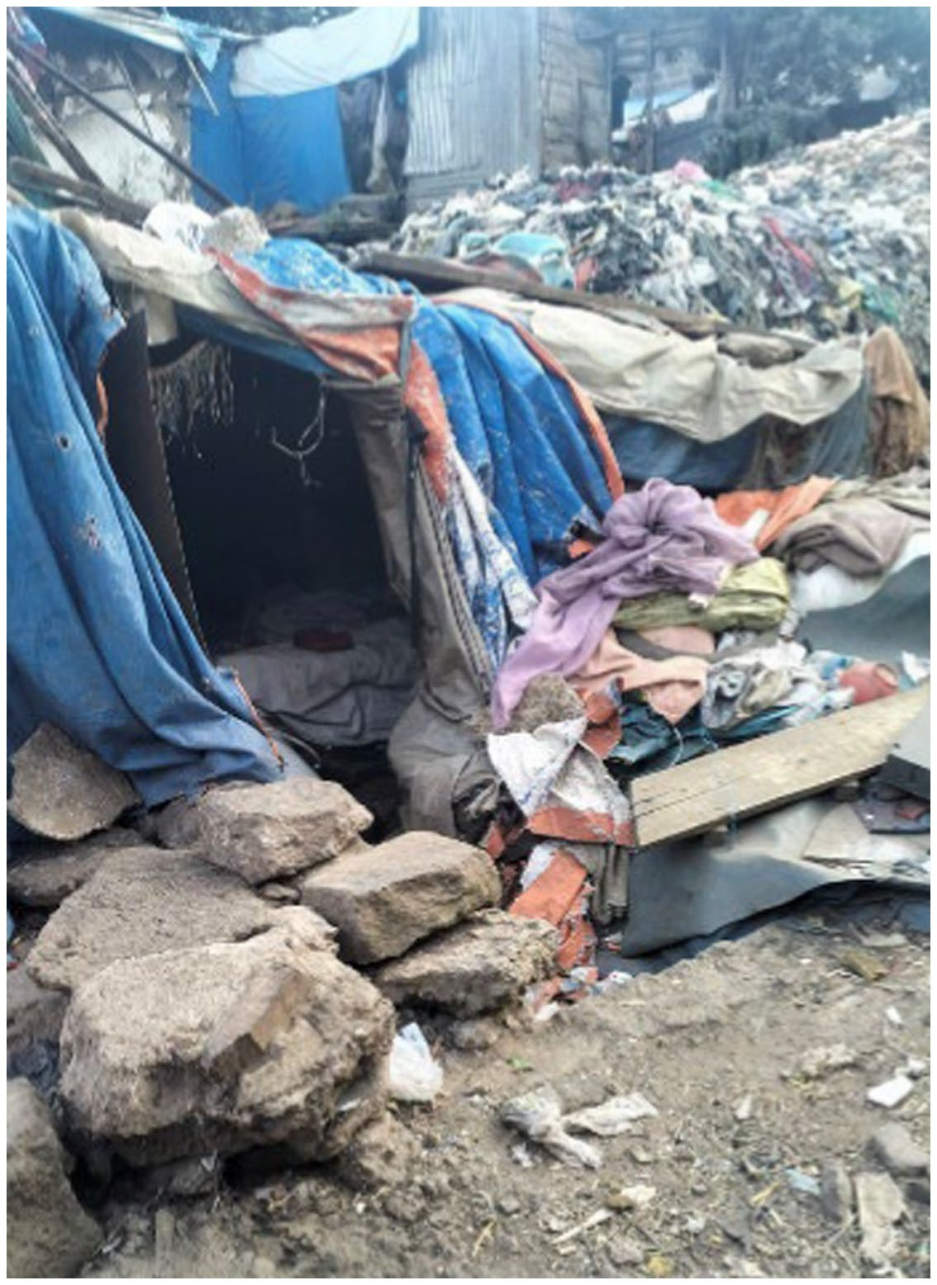
A makeshift shelter in poor condition. Addis Ababa, Ethiopia, December 2023. A homeless person’s shelter is made of stones and plastic. This is the home where individuals experiencing rooflessness reside (Image and quote credited to a 27-year-old mother experiencing street homelessness).

Although all women photographed homeless shelters, their methods of portraying the problem and their concerns differed. In some cases, they focused on the location of the shelters, which were surrounded by rubbish, to illustrate the damage that could be caused (Figure 8).

**Figure 9:**
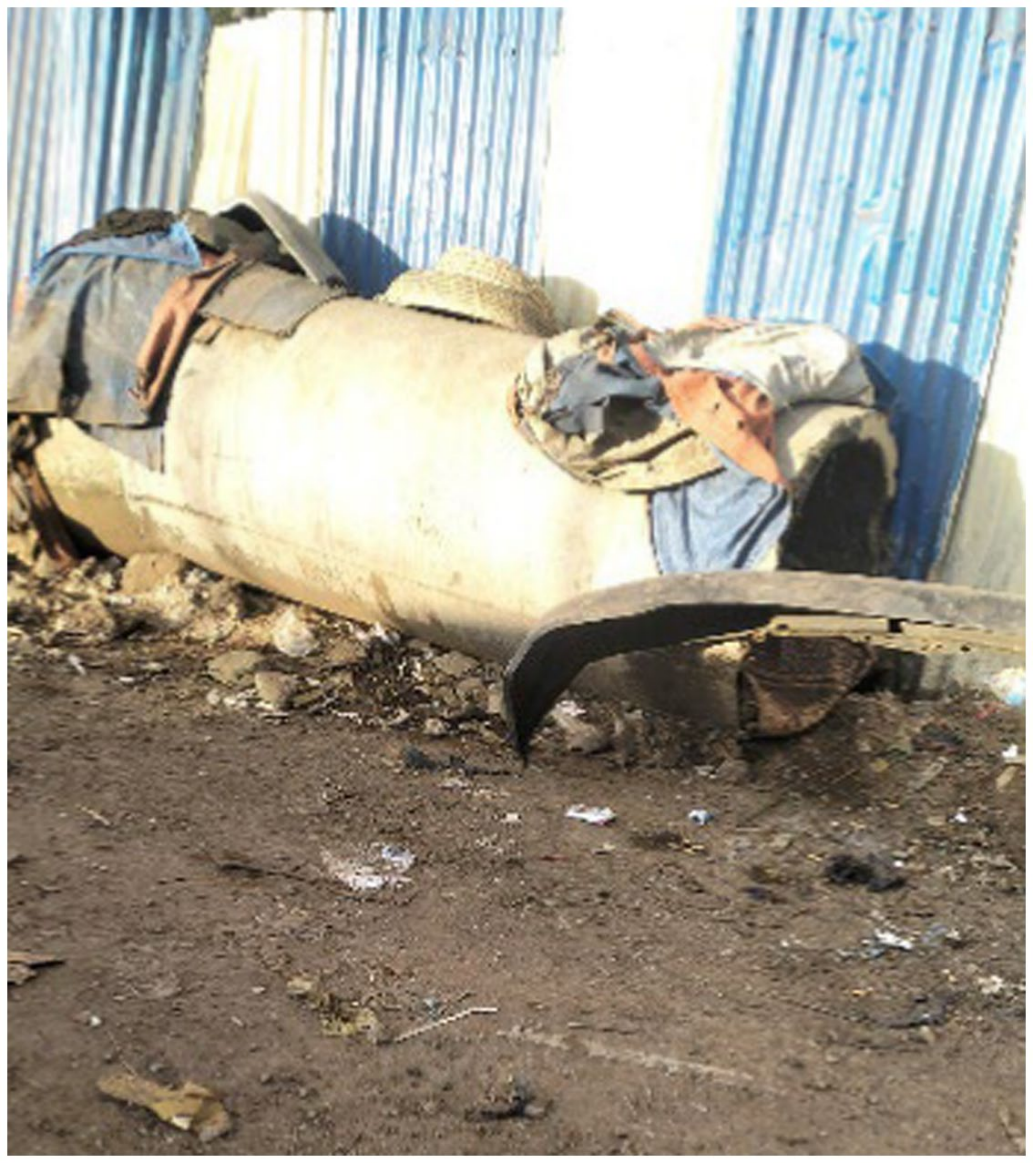
A refuge from rapists. Addis Ababa, Ethiopia, December 2023. We (people experiencing street homelessness) use these pipes temporarily when our canvas shelters (Shera Bet) are demolished. They are supported by the stones surrounding them, which prevents them from rolling. A steel pipe is attached so that approaching harassers can be heard. To avoid attacks, we hide here at night. We usually dry injera (Amharic: dirkosh-ድርቆሽ) over these pipes using a traditional basket called ‘sefed’ (Amharic: ሰፌድ) (Image and quote credited to a 28-year-old mother experiencing street homelessness).

Others described concrete pipes, which allowed them a temporary shelter when their makeshift shelters were destroyed (Figure 9).

**Figure 10:**
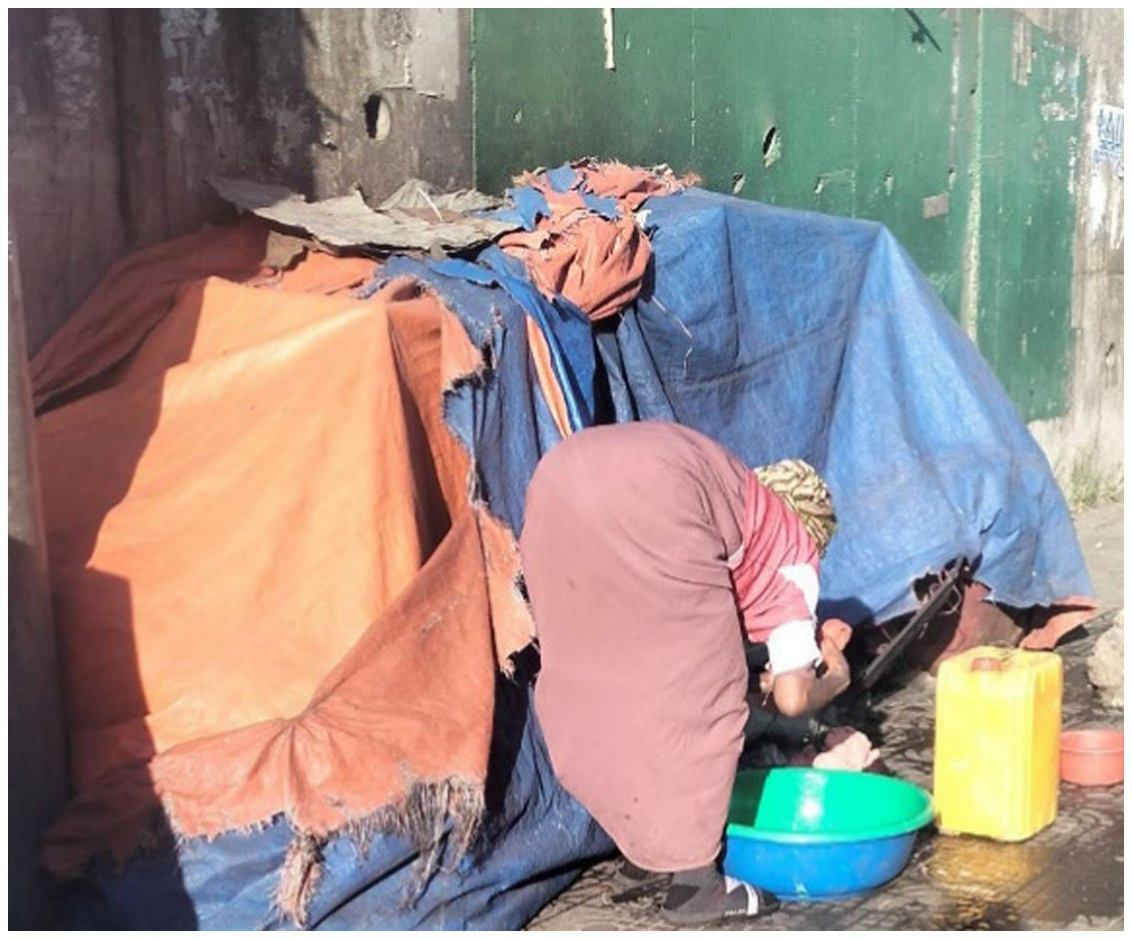
Unsafe and unsecured shelter. December 15, 2023. An unsheltered street dweller woman is washing her clothes beside her insecure shelter on the pavement between vehicles and pedestrians in Merkato’s Anwar mosque neighbourhood (Image and quote credited to a 30-year-old mother experiencing street homelessness).

Participants further described the risks faced regarding safety and security (Figure 10). The participants stressed that such a living situation could expose them to being more susceptible to rape and road traffic accidents. They also emphasised that her shelter situation could expose her to weather-related or sudden accidents.

**Figure 11:**
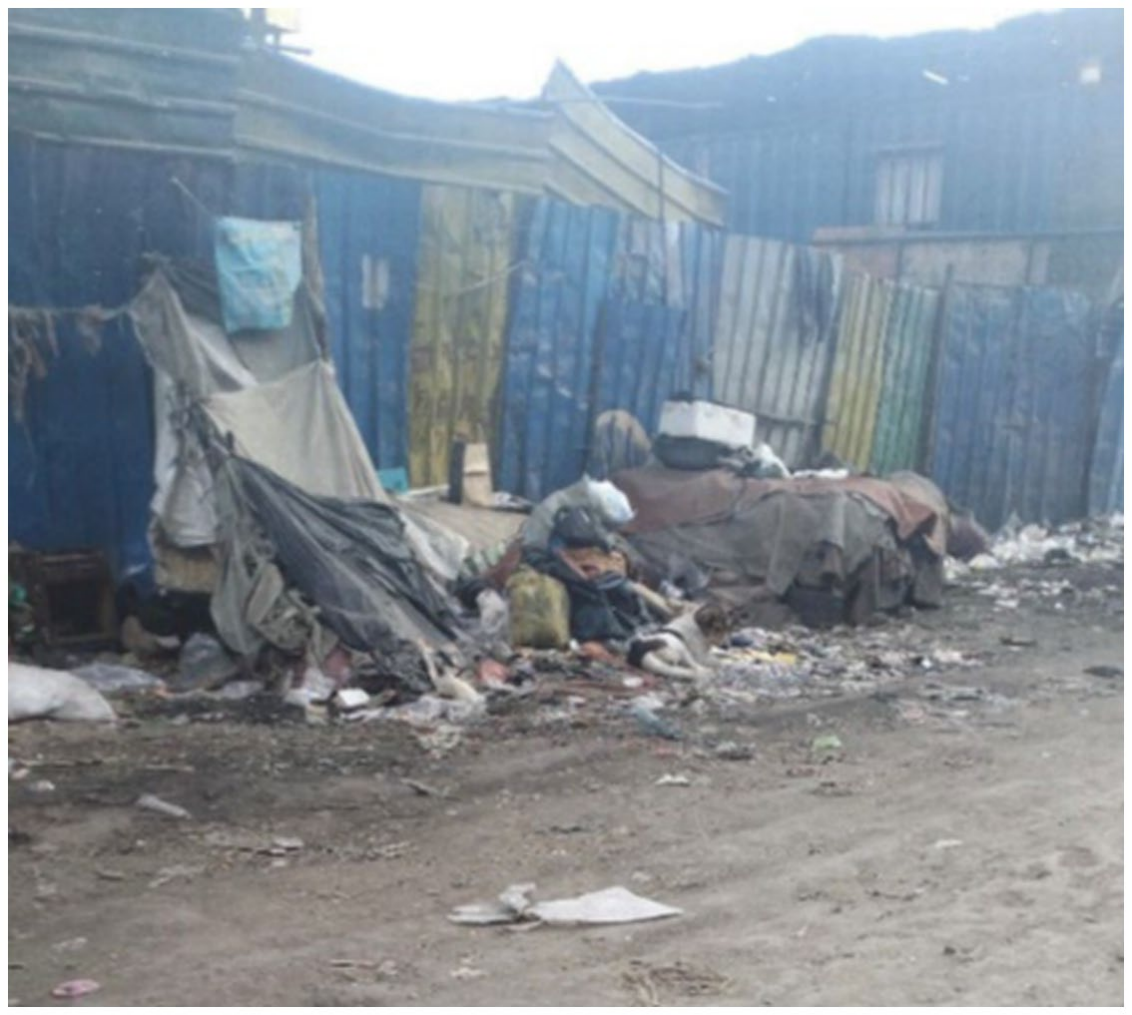
A mothers’ shelter is demolished. Addis Ababa, Ethiopia, December 2023. My home is constantly being torn down, so it is difficult to say anything about it. I mean, um… I am not interested in discussing this matter. My daughter’s friends brought us clothing in yellow plastic bags last time (Her eyes welled with tears). As our shelter was demolished, we burnt everything that remained (Image and quote credited to a 28-year-old homeless mother of three children).

Many participants reported experiencing shelter destruction in various neighbourhoods (Figure 11). Neither the attack nor the destruction of a shelter prompted the incident described to be reported. Participants perceived legal authorities as a threat instead of a source of protection.

##### 3.1.1.3 Living in a world of fear

Fear was a consistent theme across the data. Participants described various forms of fear, including the fear of losing shelters, the fear of sudden death, the fear of sleeping at night, and the fear for the safety of children. A participant questioned the meaning of protection and various sources of violence and injustice contributing to a feeling of lack of safety:

“A police officer in the Gedam neighbourhood sexually assaulted a friend of mine (a friend of the homeless woman). After she requested protection from other harassers at the police station, they assaulted her. Her mother later filed criminal charges against the rapist, but his colleagues called and threatened her. She was intimidated in an attempt to facilitate his release. No security is provided.” (P3 a 27-year-old homeless woman).

**Figure 12:**
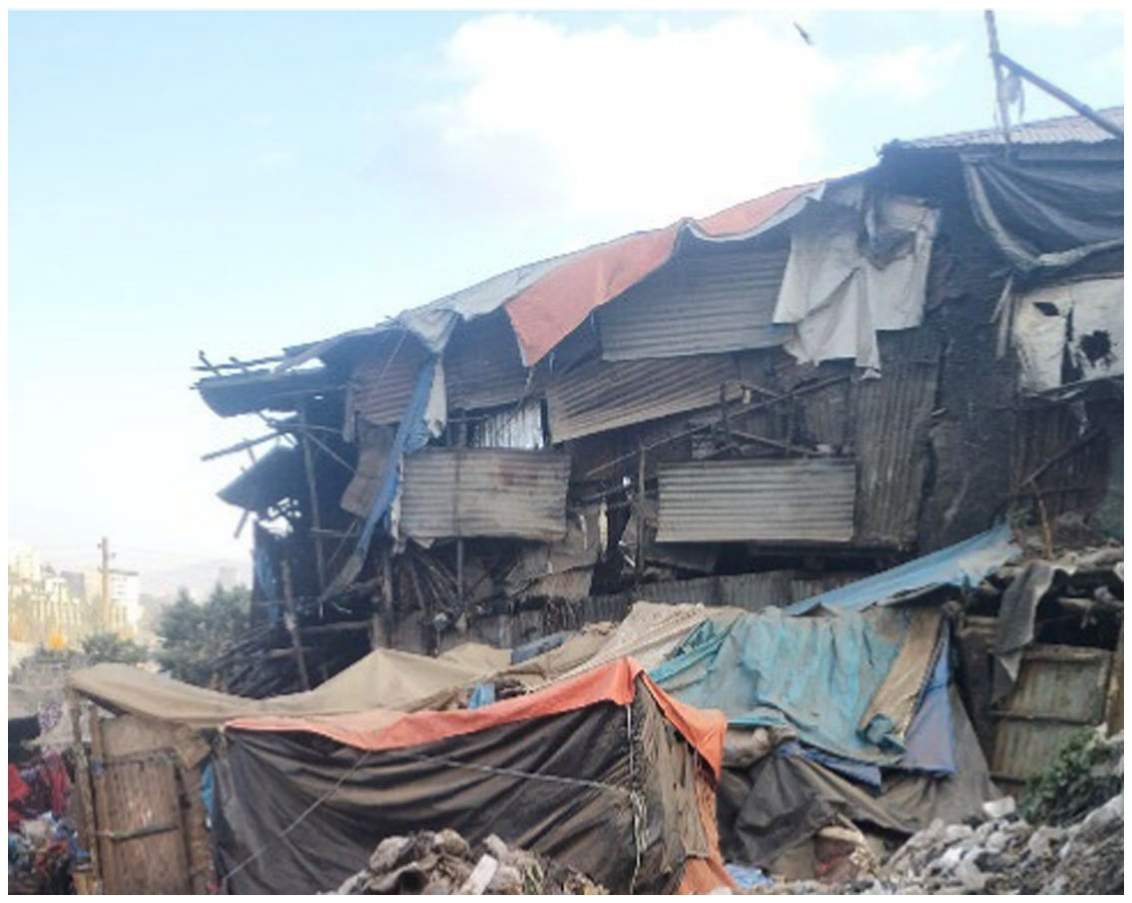
Crime Zones. Addis Ababa, Ethiopia, December 2023. These houses are rented to hide khat and other drugs, such as cocaine. They are also houses where fraudsters fabricate entirely forged documents (Image and quote credited to a mother experiencing chronic rooflessness).

The participants emphasised that not only their shelter but also their living surroundings are occupied by criminals and drug users. In addition, they mentioned that police rarely enter the area to arrest criminals and that both homeless people and locals went there for shelter and were involved in criminal activity (Figure 12).

Rather than sleeping at night, they described that they had been wandering around the town to avoid danger or hanging out with a group of other homeless individuals. Their lack of safety and security creates a permanent sense of fear.

#### 3.1.2 Theme II: experiencing dependency, shame, and seclusion while dealing with the burden of street life

##### 3.1.2.1 Substance misuse

**Figure 13:**
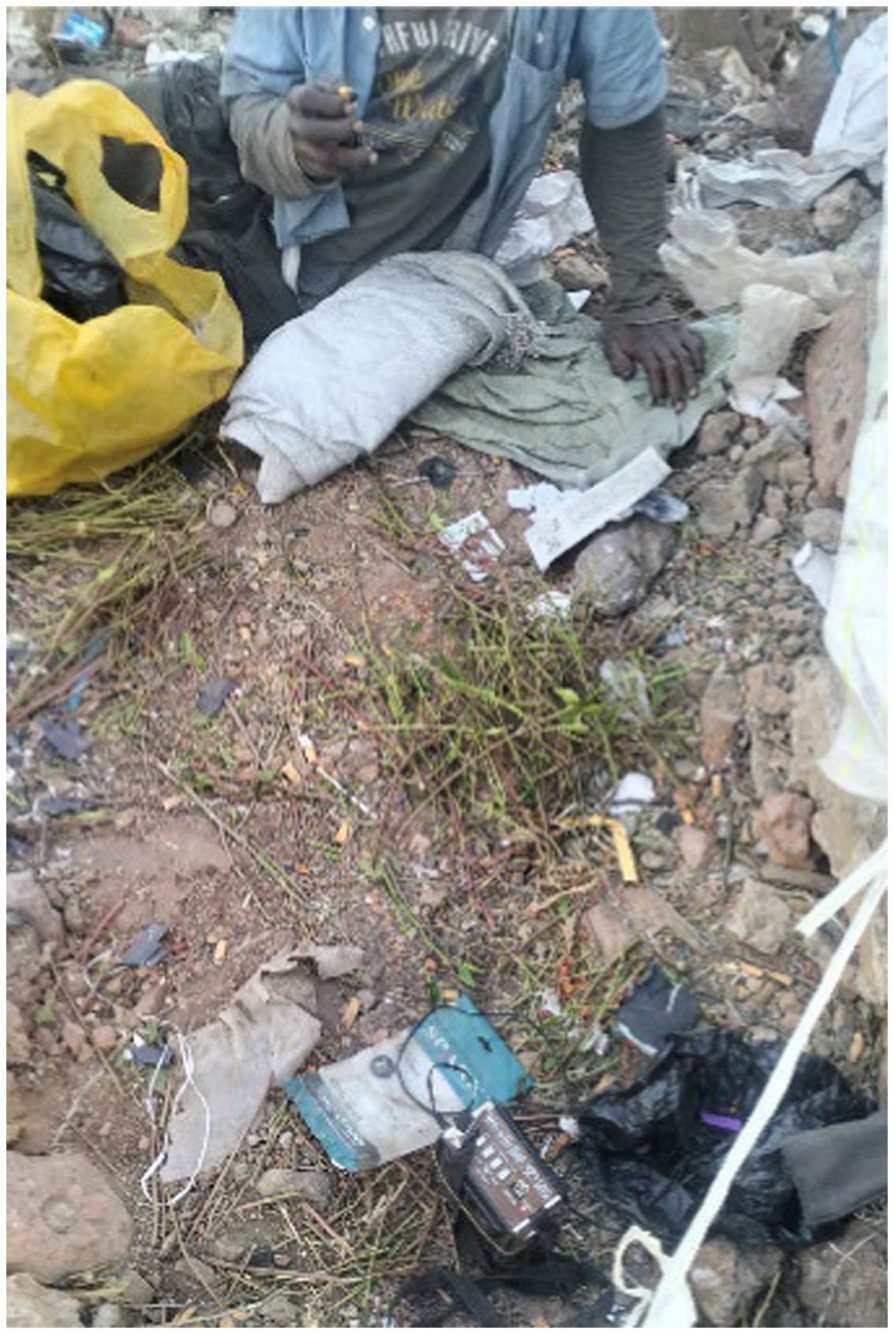
Addiction. Addis Ababa, Ethiopia, December 2023. Items visible in the photo include a khat remnant, a cigarette butt, a plastic bag, and a radio. Individuals often end up on the streets for a variety of reasons, and addiction is one of the most common factors (Image and quote credited to a 26-year-old mother experiencing street homelessness).

Multiple participants focused on photographing people with substance misuse conditions living on the streets. They shared their lived experience of using a variety of substances, such as khat, cannabis, alcohol, and inhalants, to keep awake and thereby prevent themselves from harassment. In addition to khat, cigarettes, and glue, they used other substances, such as local drinks (areke) (Figure 13).

Participants also described how substances adversely affect themselves and their children. For example:

For many years, I indulged in smoking cigarettes and chewing khat in public places. It was on the streets that my first child was born. She used to pretend to smoke by saying “Uff-(the act of smoking without actually smoking)” while I smoked. One day, while I was smoking, my child pointed to my cigarette, and I accidentally burned her hand with it. A passerby who witnessed this suggested that I give my child to the government for proper care and upbringing. But I refused, saying that I would raise her on the streets while smoking. The passerby then gave me 200 birr. Instead of using the money for my child’s food, I bought cigarettes and khat. Though I have now stopped smoking and chewing khat (A mother experiencing chronic homelessness).

##### 3.1.2.2 A lonely and isolated life

**Figure 14:**
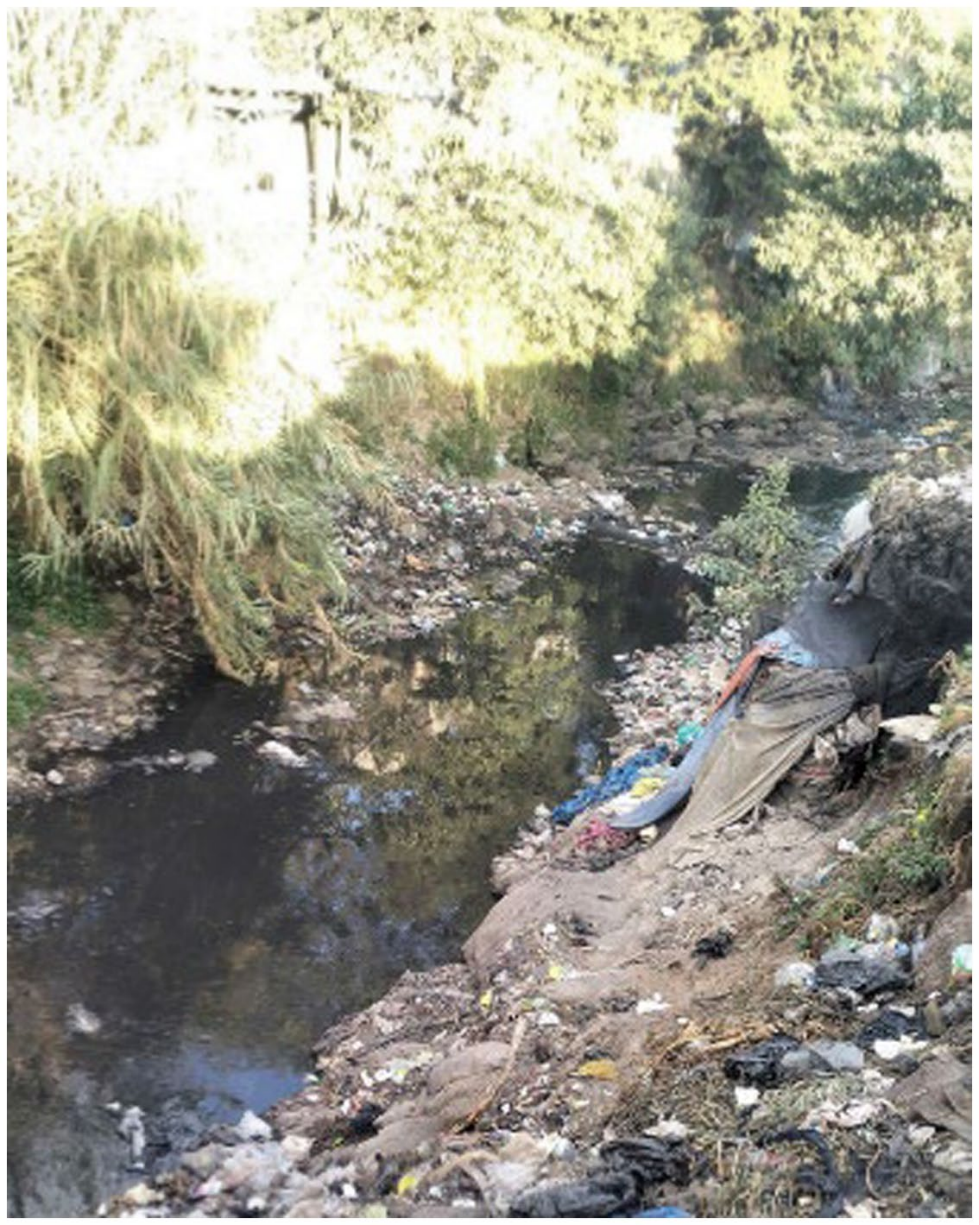
A tattered, isolated shelter. Addis Ababa, Ethiopia, December 15, 2023. A shelter is located near the river in the “American Gibi” neighbourhood. I noticed a lonely person living in the shelter by the river. Loneliness can cause one to feel distant from others. Several sources feed the river, and trash can accumulate from various locations. This presents an extensive health risk to the shelter residents. Moreover, when waste is mixed with other fluids in the river, it may cause various health problems (Image and quote credited to a 26-year-old mother experiencing street homelessness).

Some participants described how people experiencing street homelessness choose seclusion and move to isolated locations, such as river banks (Figure 14).

**Figure 15:**
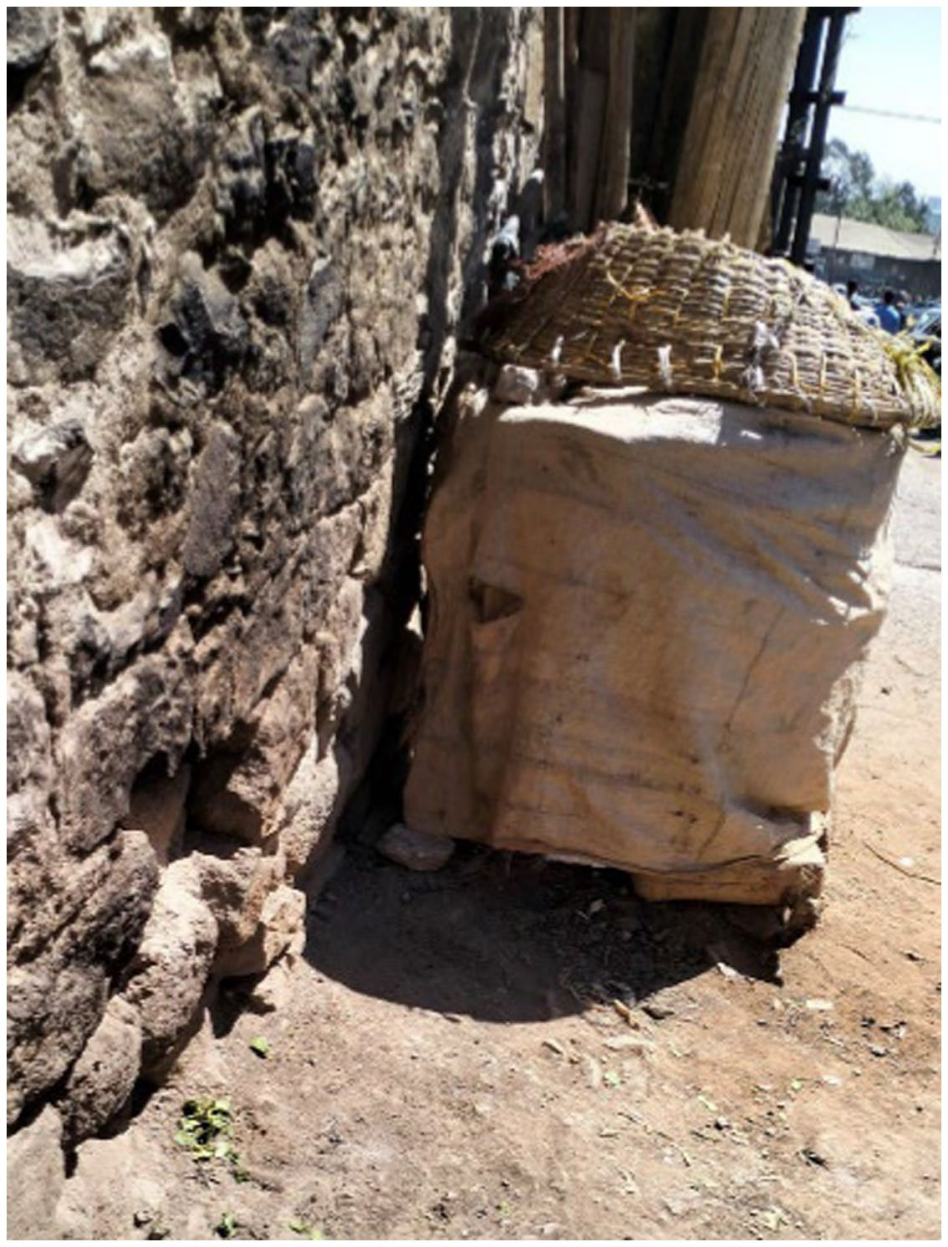
Self-isolation as a method of assault prevention. Addis Ababa, Ethiopia, December 20, 2023. There are no other shelters nearby. At first, I was curious why the woman was alone (another woman experiencing street homelessness), but then I realised I could relate to her preference for seclusion. In my experience living on the streets, I also sought out private locations for safety reasons. This was to safeguard against sexual violence (rape) (Image and quote credited to a 30-year-old mother experiencing street homelessness).

Another participant captured images of a shelter similar to the isolated shelter to depict her experience of street life. She also stressed how she managed to avoid sexual harassment (Figure 15).

##### 3.1.2.3 Everyday humiliation

**Figure 16:**
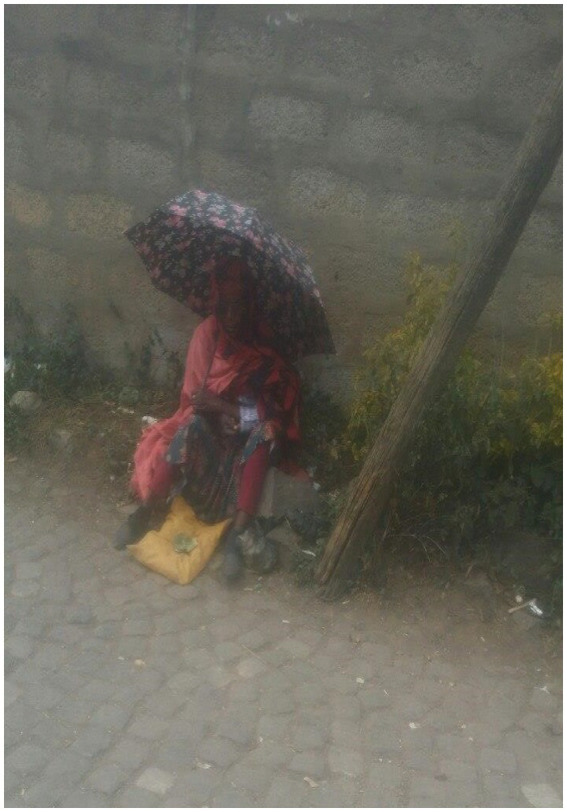
A woman begging in the street. Addis Ababa, Ethiopia, December 2023. This mother has to beg for money because of her situation. She may have kids and rent to pay. I used to beg for money in the street to buy cigarettes and khat. Her life is similar to ours (Image and quote credited to a 27-year-old mother experiencing street homelessness).

The data analysis revealed that all participants had resorted to panhandling. Participants reported that begging, common around church gates and hotel entrances, provides a livelihood for individuals of all ages and genders (Figure 16).

The women indicated that street begging allowed them to make money more quickly and efficiently than waiting in long lines for free food. A participant shared the following from her personal experience:

*Kebele workers’ (Kebele- the smallest administrative unit) families receive ten loaves of injera regularly. However, street dwellers need more access to it and often need help finding food. Homeless people spend their nights searching for food, but obtaining a loaf of injera can be challenging. The current process of waiting in line for government assistance is not helpful for us, resulting in us missing the chance to make money through begging, which we call “Shikella”- (Amharic: ሽቀላ).’ #8 a 27-year-old woman experiencing street homelessness.*

**Figure 17:**
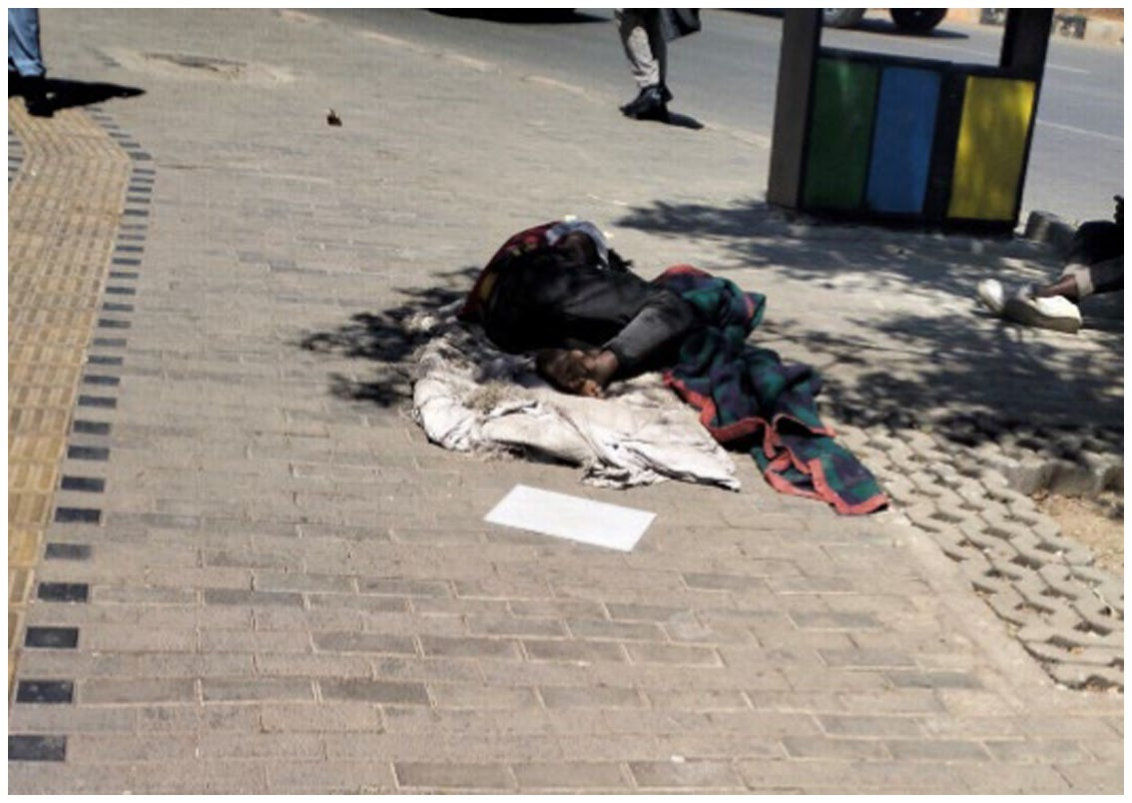
You are aware, but you do not act. Addis Ababa, Ethiopia, December 19, 2023. A homeless person lies on the street near St. Giorgis church. A paper is nearby, and it is unclear how he is doing. Throughout the city, cars and people passed by (Image and quote credited to a 27-year-old mother experiencing street homelessness).

Furthermore, in one participant’s photo, a young man lying on the streets left a note asking for assistance (Figure 17).

#### 3.1.3 Theme III: vulnerable and neglected children

Data analysis revealed that the struggles and worries of participant’s children were a major concern.

##### 3.1.3.1 Children’s hardship

Participants stressed that living on the streets is much riskier for their children than for them. A few women reported that the canvas shelter (Shera Bet) has caused young children to die at night due to its heat. Apart from adult rape, they expressed particular concern about boys and small girls. For example:

*Boys and men can also be victims of sexual assault, not just girls and women. Some organisations help victims, but I do not know their names [organisation’s name]. These organisations typically provide medical examinations and temporary housing. However, these boys display negative behaviours upon their return, which could make it challenging to approach them. Besides, associating with these boys might lead to discrimination from others (Interview #1, a 28-year-old mother experiencing street homelessness).*

**Figure 18:**
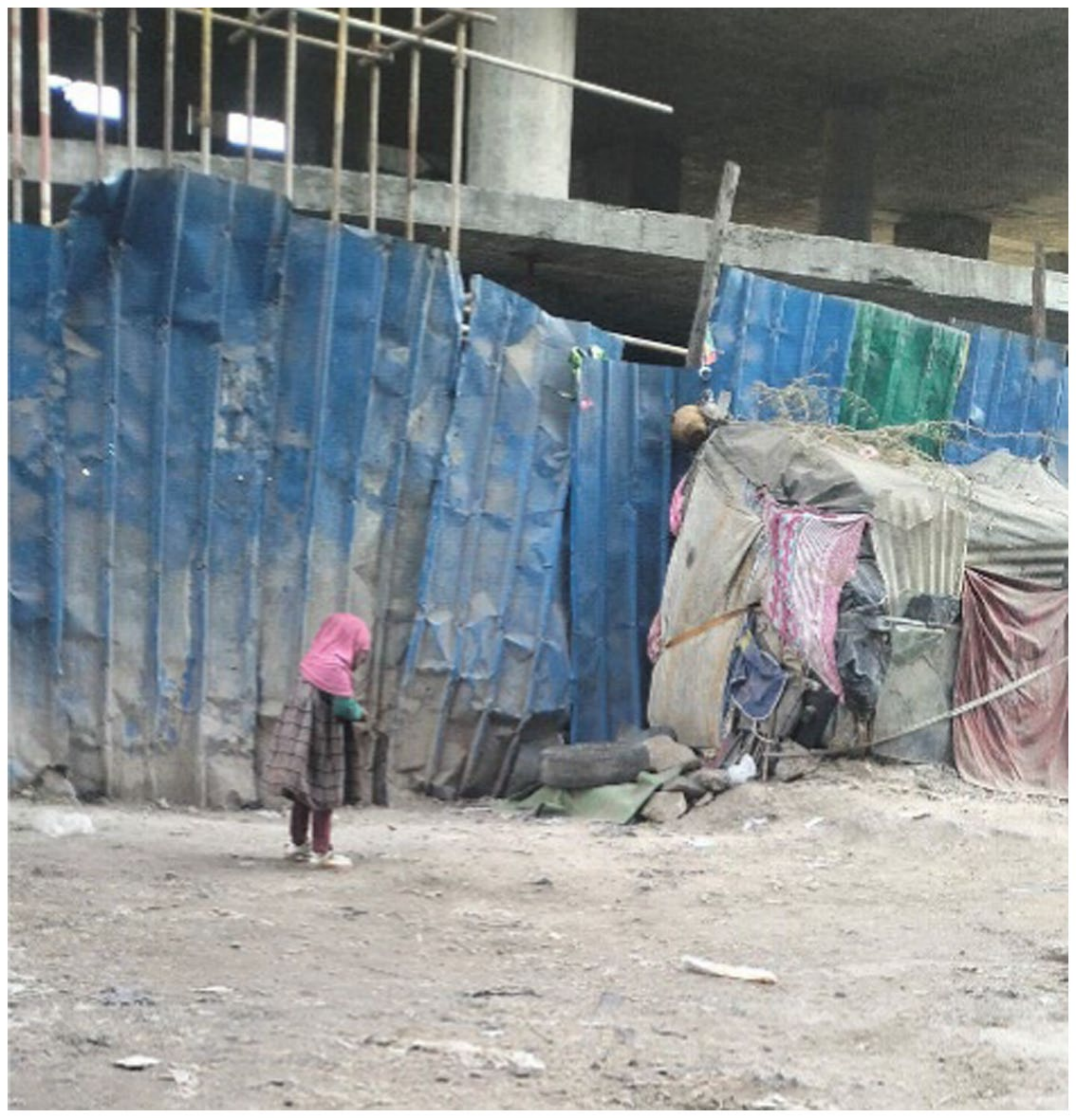
Safety risks for children. Addis Ababa, Ethiopia, December 2023. A little girl is standing next to her shelter (Shera Bet), the type of shelter homeless people live in. There is a risk of stones being thrown at the shelter if construction is built above it. This could endanger the girl’s safety. Sadly, officials often destroy the shelter without prior warning, leaving the family with nothing to live on. I took a photograph of the shelter to represent my living situation (Image and quote credited to a 28-year-old mother experiencing street homelessness).

Besides, children’s safety, which is associated with living under a construction building, was described as a concern (Figure 18).

**Figure 19:**
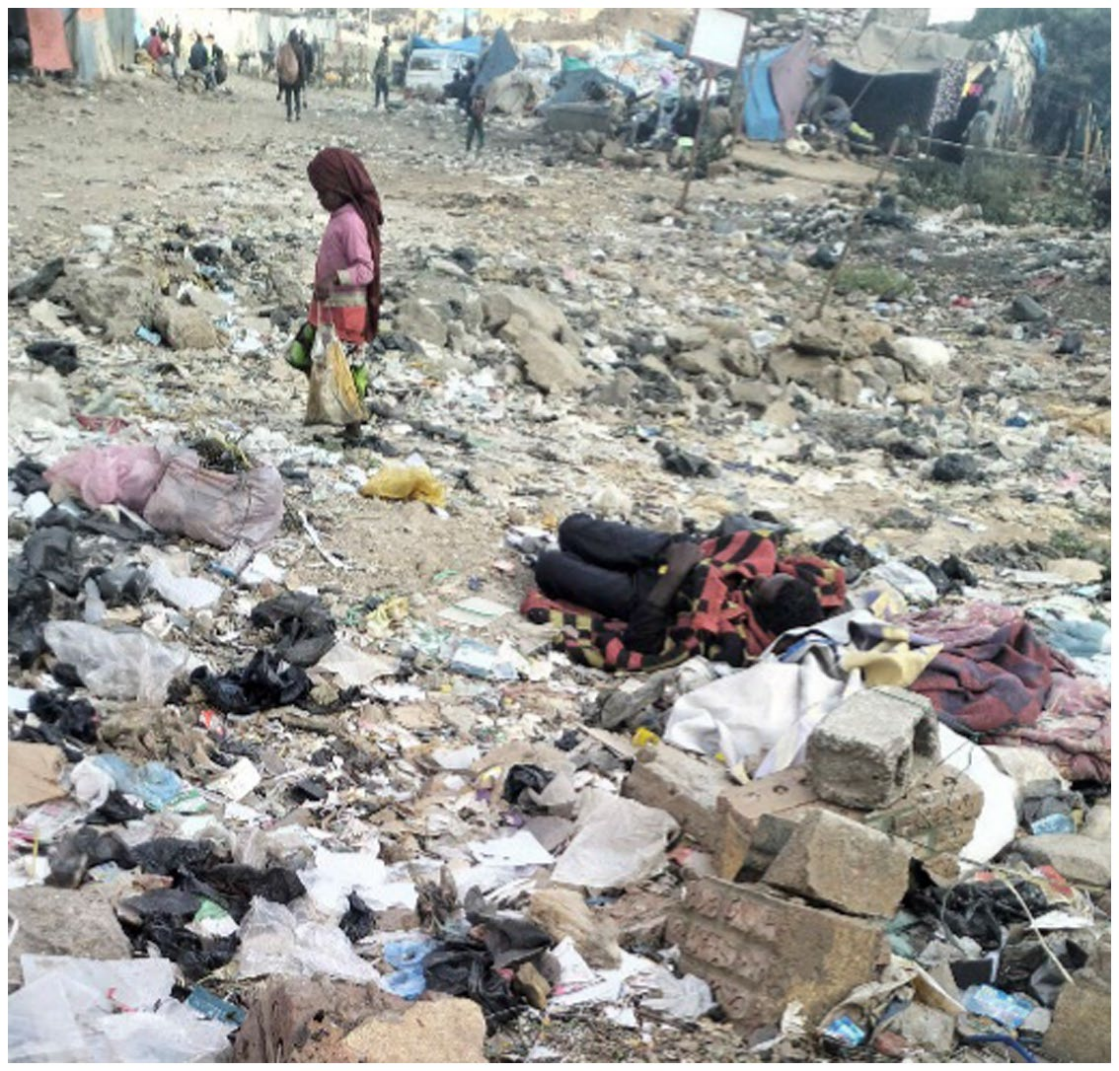
A child’s struggle with adversity. Addis Ababa, Ethiopia, December 2023. A young girl collects waste items from a landfill (Image and quote credited to a 27-year-old mother experiencing street homelessness).

Another participant photographed a child picking up waste items, explaining that street children struggle daily to survive (Figure 19).

##### 3.1.3.2 Imitating parental street life

Data analyses revealed that several mothers expressed concern about their children’s behaviour and future lives. Participants specifically discussed the psychological impact of street hardship on children. Their concerns included widespread substance misuse (khat, sniffing glue, alcohol, cannabis, as well as cigarette use) that their children observe on the streets. The participants shared their experience of observing several street children who have attempted to smoke or use other substances after they had observed their parents or other individuals using them.

##### 3.1.3.3 Barriers to early childhood education

The study’s participants voiced their concerns regarding their children’s lack of education. The requirement of providing birth certificates (usually obtained during a birth facility) for school registration poses a significant obstacle to education.

*The government or the education minister should be responsible for this issue [registration of children for education], not individuals. As a result, homeless people cannot be educated if they do not have identification documents. How can they obtain a birth certificate if they do not have a Kebele ID? (Interview #3, a 28-year-old mother experiencing street homelessness).*

The participants stressed that even though children may be able to attend school, there is a concern that they may return to a dilapidated shelter or an environment surrounded by rubbish, which may negatively affect their health and make it challenging to study.

#### 3.1.4 Theme IV: being resilient to harsh conditions

This theme highlights how participants cope in the face of harsh conditions.

##### 3.1.4.1 Normality in precarious circumstances

**Figure 20:**
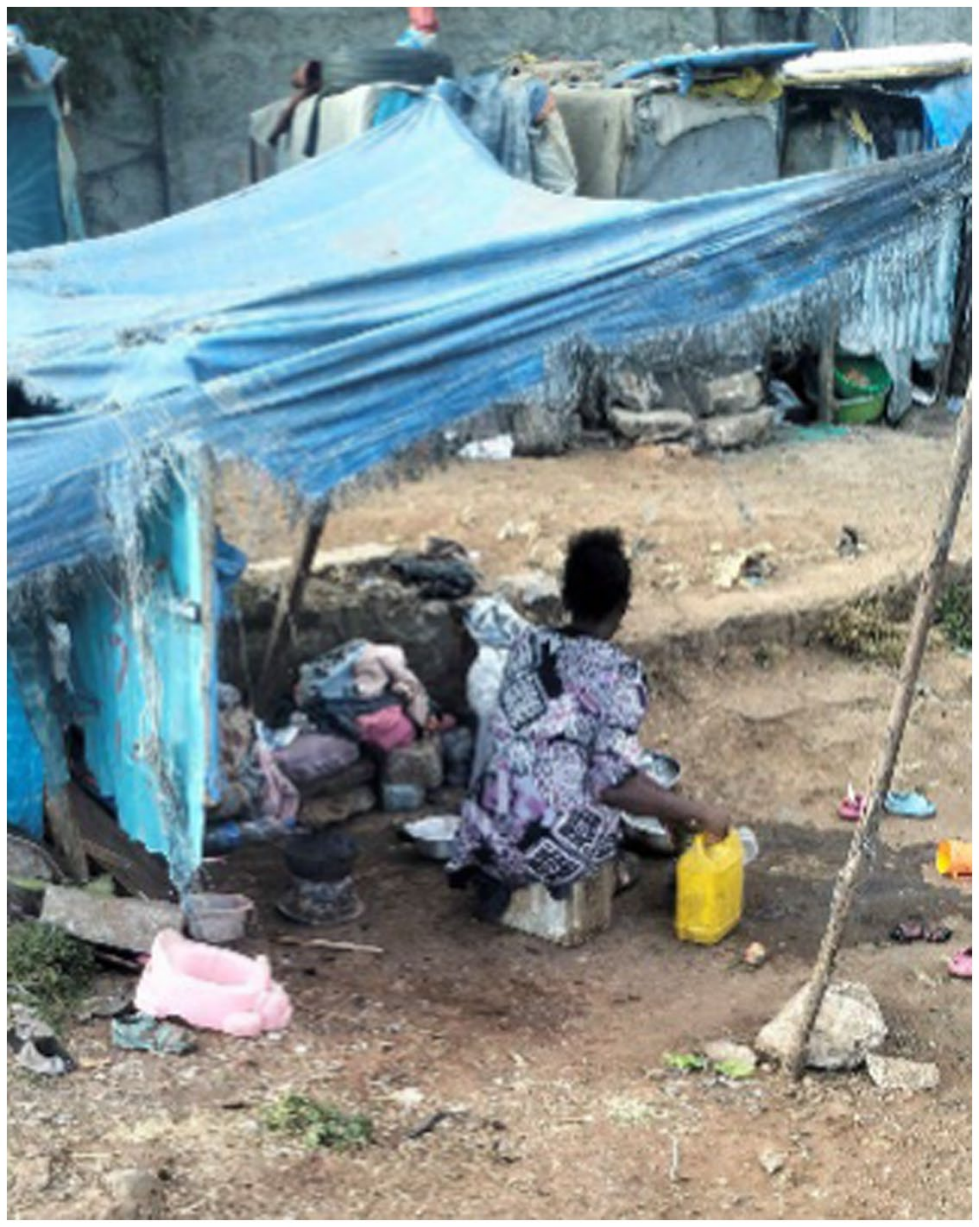
A woman carrying out her daily chores. Addis Ababa, Ethiopia, December 2023. We see a woman performing her daily chores amidst the wreckage of a destroyed shelter. Despite the difficult circumstances, she washed dishes while cooking materials and a potty were scattered nearby. As a mother, she emphasises the importance of providing her children with a healthy and clean environment. Cooking and cleaning are part of her daily routine (Image and quote credited to a 27-year-old mother experiencing street homelessness).

The participants shared their experiences, as well as those of other women, in overcoming daily obstacles on the streets. Despite being homeless, these women led their daily lives by carrying out household tasks such as washing clothes, cooking, and sending their children to school (if possible) (Figure 20).

##### 3.1.4.2 Window of hope and faith

**Figure 21:**
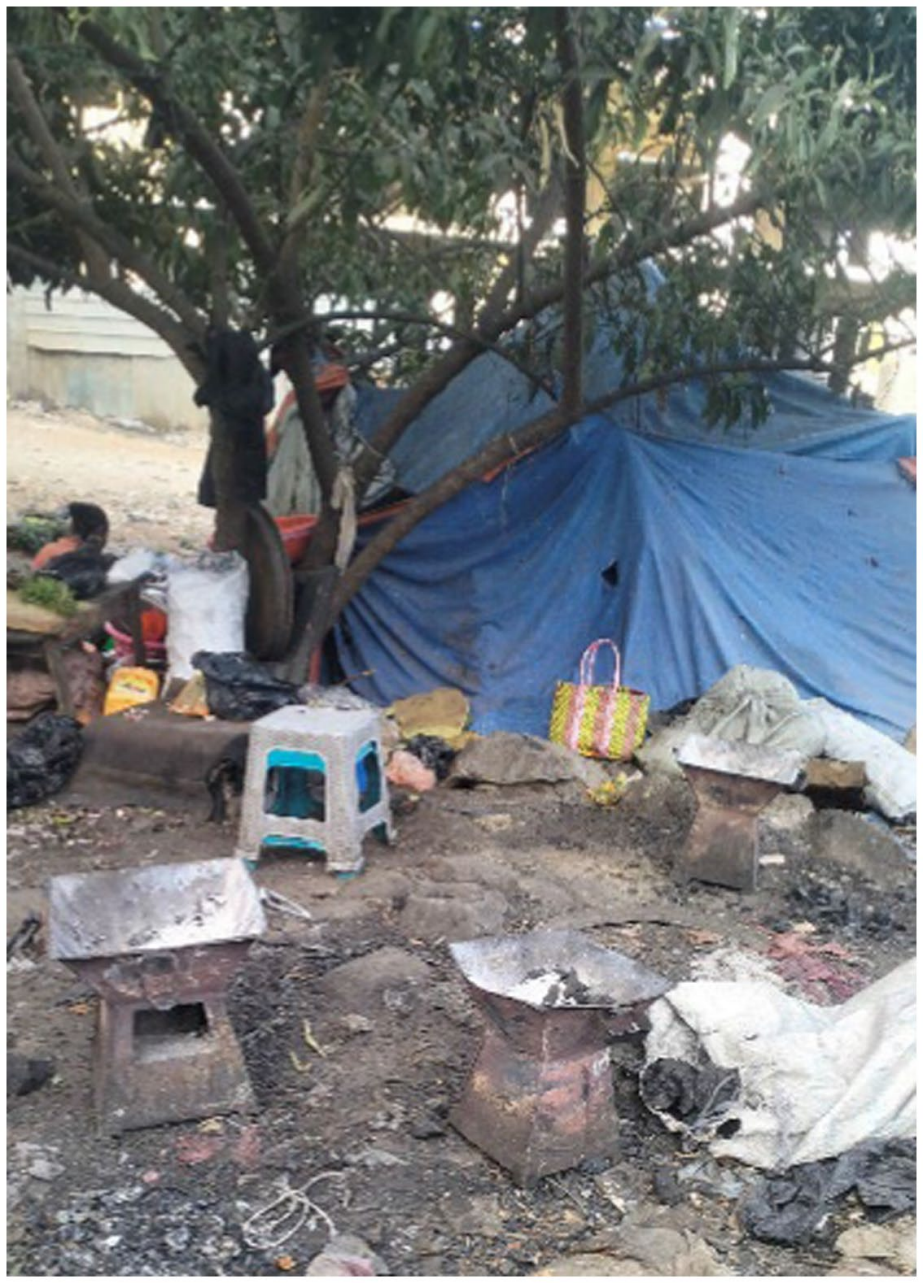
A window of hope. December 2023, Addis Ababa, Ethiopia: In this image, a homeless woman prepares leftover foods and sells them to others experiencing rooflessness. She purchases and resells leftovers from different sources as part of her business. It is evident from the photograph that the woman has worked hard and is strong (Image and quote credited to a 30-year-old mother experiencing homelessness).

The participants expressed the hope that once they have completed their training at BICIDO, they can be independent through their businesses. Participants described stories of women starting small businesses despite their struggles. A participant photographed one of these women selling leftovers to others experiencing street homelessness (Figure 21).

##### 3.1.4.3 Social cohesion

**Figure 22:**
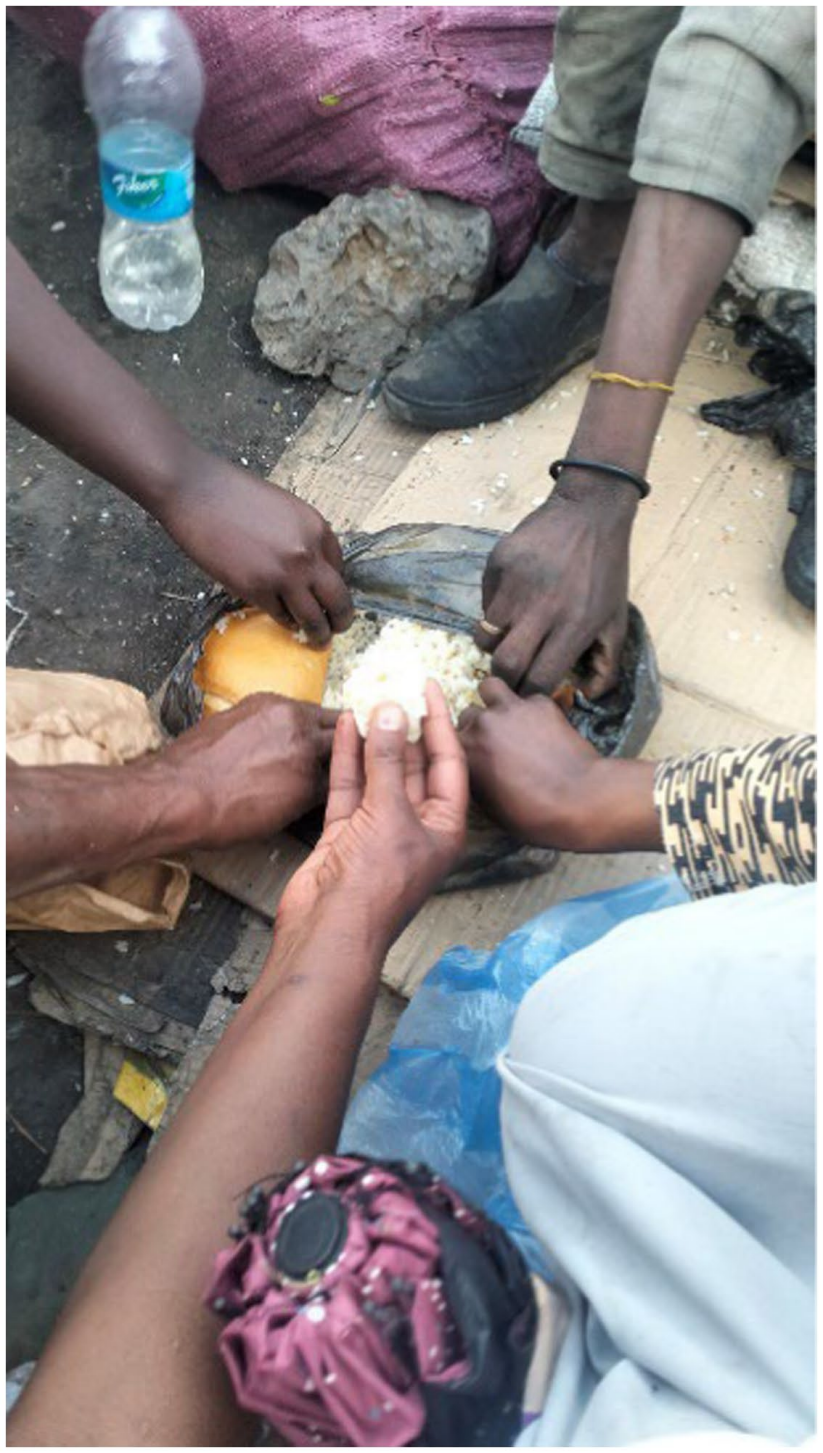
Cohesion. Addis Ababa, Ethiopia, December 2023. Here, a group of individuals experiencing rooflessness eat rice from leftover foods (called “bulle”). This is how we survive on the streets. This photograph shows our culture’s unity and how we eat food. Even though we all behave differently on the streets, there is a sense of caring among us. To live peacefully with people requires much tolerance, and we bring leftovers in the “bulle” container for later use whenever we share a meal (Image and quote credited to a 26-year-old mother experiencing street homelessness).

The participants discussed their strong social support network while living on the streets. They indicated mutual support through communal eating, group living, trying to take care of themselves and each other, protecting their friends, sharing shelters when someone’s shelter was destroyed, and supporting a person in danger as a part of their daily lives. A participant’s image is one of many photos they took to demonstrate their social connection (Figure 22).

## 4 Discussion

This study aimed to describe and analyse women’s experiences of street homelessness in their own terms and their suggestions to address their unmet needs. Participants described a multitude of unmet basic needs and security concerns. The analysis identified four major themes: “deprivation of basic needs,” “experiencing dependency, shame, and seclusion while dealing with street-life burdens,” “the vulnerability and neglect of children,” and “being resilient to harsh conditions.”

Internationally, the state parties have agreed to recognise the right to an adequate living standard, including access to food, water, and shelter, in Article 11 of ICESCR (42). However, all participants described a lack of access to basic needs (physiological and safety needs).

Studies from Ethiopia (43), Eldoret town in the western region of Kenya (44), Bonn, Germany (45), Ann Arbor, Michigan (46), and California (47) have supported these results, showing that people experiencing homelessness have critical unmet physiological necessities. This leads to the impression that women experiencing street homelessness have unmet basic needs in all parts of the world. Economically developed and low-income countries have yet to provide adequate living conditions for individuals experiencing homelessness.

In addition to the difficulty of accessing food and water, women experiencing homelessness in this study reported a lack of shelter and constant security threats. Researchers from Ethiopia (43), central Auckland, New Zealand (31), and Seattle, Washington (48) have reported similar findings.

Homeless individuals are frequently victimised by police officers and controllers who destroy their makeshift shelters, causing them great distress in the study area. Additionally, the destruction of their shelters has heightened their vulnerability to violence. However, in Ethiopia, illegal occupants are prohibited from sleeping or constructing shelters in public places, and the construction and land use regulations and security regulations provide authorities with the legal framework to take action as needed (49). As stated in the Re-Enactment of Urban Lands Lease Holding Proclamation (Proclamation no. 272/2002) (49), Article 16 (2), “The appropriate body may clear urban land with a property thereon from illegal holders by serving a written notice warning the person having illegally held it to move away and without any need of giving him clearance order and compensation following this Proclamation.” In addition, Article 20 (3) stated that “The appropriate body may order the police wherever it finds it necessary to use force pending its receipt of land.” The finding of this study is similar to a study that reported the invasion of privacy of people experiencing homelessness (50) and the criminalisation of homelessness (51).

Women in this study stressed living in a constant state of fear, experiencing sleepless nights as a result of their fear of violence, victimisation, and potential risks in public spaces during the night. This aligns with the findings of a study in Brisbane, Australia (52), which highlighted the specific risks associated with difficulty sleeping due to worries about personal safety and the potential for rape. Other studies from India (53, 54) portrayed street life as surviving in a physically and mentally challenging environment. Also, Broll et al. (55) reported that violent victimisation increases women’s likelihood of experiencing multiple episodes of homelessness.

The participants expressed concern that people experiencing street homelessness often live in abandoned, waste-filled areas, which can severely threaten their health and safety. Furthermore, they described a scarcity of accessible locations and personal circumstances that forced some individuals to live on riverbanks teeming with waste (both liquid and solid) and lacking proper drainage. The result of this study is in agreement with those reported by studies conducted in Delhi (56), Arizona (57), Tokyo (58), and Tennessee (59). The authors also highlighted the health risks associated with living in such a precarious environment that could adversely affect a homeless individual’s health in numerous ways (56, 60).

In addition to the deprived basic needs, participants also discussed the misuse of substances (stimulants, depressants, and hallucinogens) on the streets (in some instances, among people who display disorganised behaviours and poor self-care) and the use of these substances to keep awake to avoid attacks. It has been observed that some of them had long-term substance misuse or used substances uncontrollably despite harmful consequences before they achieved “overcoming addiction,” often after observing their children mimic their behaviour. We find Bandura and Walters (61) theory of social learning, which highlights learning behaviour through modelling, helpful in making sense of the women’s and their children’s experiences. Children of women experiencing rooflessness may pay close attention to how others behave and subsequently apply it themselves (62). As a result of modelling their parents’ behaviour, many children consume substances that may have complicated effects on them, mainly if they are led to believe that substance use is a valuable means to cope with harsh weather conditions and night-time sexual assaults.

A combination of negative and positive attitudes towards begging was reflected in the participants’ experiences. Many participants identified begging as a way to support themselves and as a business (“shikella”) due to a lack of employment and government assistance, echoing existing research from Ethiopia (63), Johannesburg (64), and Paris (65).

Existing studies also revealed begging as a response to poverty (63), societal oppression, and prejudice (66). This results in feelings of demeaning treatment, hatred, depression, and an identity crisis within a given country (66). In addition to begging and substance misuse, the women mentioned avoidant coping strategies (e.g., withdrawal and isolation) as coping mechanisms. One study confirmed this finding and asserted that isolation is a predictor of mental health problems (67).

The findings of this study highlight that children living on the streets are particularly vulnerable and neglected. A Gondar, Ethiopia study has supported this finding (68). Children experiencing rooflessness are one of the most vulnerable groups in society. Even though the transformative agenda, which aims to achieve the foremost promise of leaving no one behind, relates to them through 48 SDG indicators (69), they still suffer from a variety of mental and physical health conditions, inequities, social exclusion, economic hardships, safety issues, and even survival concerns (70). Although the UN SDGs have given special attention to children, according to a recent phenomenological study (71), street children in Addis Ababa, Ethiopia, are encountering widespread challenges like social network fragmentation, a lack of legal protection, child trafficking, harassment, and a shortage of necessities.

Finally, women with dependent children described coping strategies for dealing with and adjusting to the adverse and stressful situations of street life. The findings are consistent with studies showing resilience and an optimistic view of the future among homeless women, which promotes the formation of social networks, a sense of hope, and a readiness to move forward (8, 17, 72).

### 4.1 Proposals for change by homeless women and the benefits of the project for them

Incorporating women’s voices through photographs, storytelling, and group discussions, the photovoice approach will enable researchers and participants to engage meaningfully with issues concerning street homelessness. This kind of consideration and inclusion will encourage people experiencing homelessness to participate in the research process, develop their ideas in the context of homelessness, and share their experiences (27). Being part of a research team makes homeless people feel like they are essential to society and helps them maintain good self-esteem. In this study, the photovoice participants described not only their living conditions but also their proposals for change, hoping that responsible policymakers, officials, and stakeholders would consider them. This shows how this method encourages individuals experiencing homelessness to consider their potential and feel empowered to advocate for others, which may positively impact their self-perception (28).

The women arrived at several thoughtful suggestions and proposals for how governmental and non-governmental services could improve ongoing services. These change proposals mainly focused on housing access, food and water security, job opportunities, and addressing street children’s hardships and the unmet needs of these hard-to-reach population groups in healthcare, education, legal protection, and social support. Below are the suggestions for change provided by women participating in the photovoice study who were asked to give ideas to improve the lives of women experiencing homelessness in Addis Ababa:

- The provision of a house is the crucial and primary issue for us. With a stable home, we can do everything else: work and make money to support ourselves.
- It would be good if the government could provide housing and food for us. We suffer from hunger, a lack of shelter, a polluted environment, and cold weather.
- Employment opportunities should be made available for individuals experiencing homelessness who have shown dedication to surviving their complex street lives.
- Education and advice regarding the effects of substance misuse should be provided to individuals experiencing homelessness.
- Justice should be available to those who have suffered violent attacks.
- The government should provide street dwellers with ID cards and arrange for birth certificates for those children born on the streets.
- Living in a community or close to other individuals would be more beneficial than in a secluded situation.
- The government should support street dwellers’ education, particularly the Ministry of Education.
- Kebele workers should provide free food to street dwellers in a fair and timely manner, and government officials should personally visit the streets and food distribution sites to assess the situation rather than just read about us and look at our photos.
- Immediate support should be provided for those individuals with disabilities and medical issues who are sleeping on the streets.
- Medical and social support should be offered to individuals struggling with substance abuse.
- Addis Ababa should be made a clean and attractive place by providing shelter and support for those experiencing homelessness, especially given its status as the African Union’s seat (A woman born and raised in Addis Ababa who attended secondary school suggested this).

### 4.2 Policy and practical implications of the study

Following the study results and participant recommendations, the government needs to adopt the Housing-First model in Addis Ababa, Ethiopia. This program has proven effective in addressing homelessness in many countries and includes it in programs for urban disadvantaged groups (73). The Housing First program encompasses most of the recommended areas. The central principles of this approach address several aspects of a person’s life, including housing as a human right, giving service users choice and control, separating housing and treatment, recovery orientation, harm reduction, active engagement without coercion, person-centred planning, and flexible support (74). Adopting this model as a national strategy and implementing it in a coordinated fashion could help break the street life cycle (75). In this approach, the individual is not dominated by the provider’s approach of “This is what you need” but rather by their voice of “This is what I need.” This is similar to the photovoice approach we used, in which we listened to what participants had to say, such as, “These are the suffering I have endured that requires immediate attention.” This approach allowed us to ensure their voices were heard.

Similarly, in the Housing First model, client-centred and need-based support are the central approaches to client intervention. When an individual participates in the decision-making process, the outcome can improve. Indeed, the Housing First model’s social engagement principles have the potential to empower women experiencing homelessness and enable them to be accepted by the community. As mentioned above, the women also suggested facilitating recovery through rehabilitation. This model’s harm reduction and recovery orientation principles include trauma-informed care and integrated rehabilitation to help people who are recovering from addiction and have been through traumatic events (75). Their suggestions can be made effective through proper delivery and coordination with institutions specialising in mental health and addiction care and support, such as St. Amanuel Mental Specialized Hospital, Geferssa Rehabilitation Center, New Life Rehab Center, the Mental Health Service Users’ Association (MHSUA) of Ethiopia, and ongoing homeless-focused governmental and non-governmental project implementers. There is a need for significant attention and budget allocation from several ministries for this model to be effective and have some long-term and sustainable impact. Among these are the Ministry of Women and Social Affairs, the Ministry of Urban Development and Construction, the FDRE Policy Studies Institute (PSI), UNICEF, the Ministry of Labor and Skills, and the Addis Ababa City administration.

### 4.3 Limitations

The participants are limited to women experiencing street homelessness in Addis Ababa. We included only women from BICDO, where women were admitted to the organisation from various neighbourhoods in Addis Ababa. This is a partial representation, as we focused solely on one organisation. Furthermore, this sample included only women with children, which can affect the view of street life because perspectives may vary among women with dependent children, never-married women, and single women. This study did not include women not involved in any programs or interventions, which may hinder the ability to obtain a complete picture of street life.

## 5 Conclusion

To conclude, the results of this study demonstrate that women and their children experiencing street homelessness face an array of adversities. Despite all these challenges, the results demonstrated the ability for optimistic adaptation in the face of misfortune and perseverance seen in street life, which is a glimmer of hope. Since all participants were mothers of dependent children, their attention was drawn to children experiencing homelessness, demonstrating that children are also left behind and unprotected. To our knowledge, this is the first photovoice study about women experiencing street homelessness in Ethiopia. Despite some limitations, this study contributes considerably by enabling women experiencing homelessness to show street life on their terms, promote active dialogue, advocate for their lives and living circumstances, and serve as a voice for others experiencing homelessness. This further helps develop interventions to help address their homelessness. Researchers should include various groups of people experiencing homelessness, as well as legal bodies, to enable an investigation of how government bodies, different age groups, childless women, and genders see their street lives differently.

### 5.1 Ethical considerations

We obtained ethical approval for this study from the Institutional Research Ethics Review Committee of the Addis Continental Institute of Public Health (ACIPH), the Addis Ababa City Administration Health Bureau (AAHB) (Registration numbers ACIPH/IRERC/005/2023 and A/A/7214/227), and the Swedish Ethical Review Authority (Dnr 2024–05261).

We obtained a permission letter from the Addis Ababa Labour and Social Affairs office and the BICDO to conduct the study. Additionally, we adhered to the International Ethical Guidelines for Biomedical Research with Human Subjects.

KY read a translated Amharic information sheet to all participants; she also read the consent form during the interview and recruitment to those women who could not read and write. We obtained all participants’ written consent before the photovoice work.

We took all necessary measures to protect the participants’ privacy throughout the study, minimising risks. We conducted interviews privately to respect the participants’ confidentiality; no identifiable information was included. We informed the participants that they could withdraw at any time. We trained the women to avoid dangerous spaces or situations during fieldwork. Permits were not required to photograph environments and public settings; however, we instructed the women not to take pictures that could harm others’ reputations, safety, and rights. In addition, we put procedures in place to ensure ethical photography (no faces). Hence, we did not include photos that showed faces for further analysis.

## Glossary Amharic terms

**Table:** 

Areqe (Amharic: ዐረቄ)	“An Ethiopian distilled liquor similar to katikala. When made by a distillery rather than at home, it is flavoured with anise, essentially an Ethiopian ouzo.”
Bulle (Amharic: ቡሌ)	Leftover food
Injera (Amharic: እንጀራ)	“The Ethiopian traditional flatbread is made from a fermented batter and cooked on a large round surface called a mitad. The bread is smooth on the bottom, bubbly on top, and has a spongy consistency.”
Dirkosh (Amharic: ድርቆሽ)	“Sun- or oven-dried pieces of injera, usually crispy and crunchy”
Sefed (Amharic: ሰፌድ)	“A traditional basket with an upward curve made of dyed grass straws and palm leaves”
Shikella (Amharic: ሽቀላ)	“The linguistic root of shikella may be traced to the Arabic shighul, which means “work.” A collective term for a wide variety of activities that people carry out in the informal sector as sources of livelihood” (64).

## Data Availability

The datasets used and analysed during the current study are available from the corresponding author upon reasonable request.
